# A global ocean atlas of eukaryotic genes

**DOI:** 10.1038/s41467-017-02342-1

**Published:** 2018-01-25

**Authors:** Quentin Carradec, Eric Pelletier, Corinne Da Silva, Adriana Alberti, Yoann Seeleuthner, Romain Blanc-Mathieu, Gipsi Lima-Mendez, Fabio Rocha, Leila Tirichine, Karine Labadie, Amos Kirilovsky, Alexis Bertrand, Stefan Engelen, Mohammed-Amin Madoui, Raphaël Méheust, Julie Poulain, Sarah Romac, Daniel J. Richter, Genki Yoshikawa, Céline Dimier, Stefanie Kandels-Lewis, Marc Picheral, Sarah Searson, Tara Oceans Coordinators, Silvia G. Acinas, Emmanuel Boss, Michael Follows, Gabriel Gorsky, Nigel Grimsley, Lee Karp-Boss, Uros Krzic, Stephane Pesant, Emmanuel G. Reynaud, Christian Sardet, Mike Sieracki, Sabrina Speich, Lars Stemmann, Didier Velayoudon, Jean Weissenbach, Olivier Jaillon, Jean-Marc Aury, Eric Karsenti, Matthew B. Sullivan, Shinichi Sunagawa, Peer Bork, Fabrice Not, Pascal Hingamp, Jeroen Raes, Lionel Guidi, Hiroyuki Ogata, Colomban de Vargas, Daniele Iudicone, Chris Bowler, Patrick Wincker

**Affiliations:** 1 CEA - Institut de Biologie François Jacob, Genoscope, Evry, 91057 France; 2 CNRS UMR Metabolic Genomics, Evry, 91057 France; 3 Univ Evry, Evry, 91057 France; 4 Institute for Chemical Research, Kyoto University, Gokasho, Uji, Kyoto 611-0011 Japan; 5 Department of Microbiology and Immunology, Rega Institute, KU Leuven, Herestraat 49, Leuven, 3000 Belgium; 6 VIB Center for Microbiology, Herestraat 49, Leuven, 3000 Belgium; 7 Ecole Normale Supérieure, PSL Research University, Institut de Biologie de l’Ecole Normale Supérieure (IBENS), CNRS UMR 8197, INSERM U1024, 46 rue d’Ulm, Paris, F-75005 France; 8 CNRS, UMR 7144, Station Biologique de Roscoff, Place Georges Teissier, Roscoff, 29680 France; 9 Sorbonne Universités, UPMC Univ Paris 06, UMR 7144, Station Biologique de Roscoff, Place Georges Teissier, Roscoff, 29680 France; 10 Structural and Computational Biology, European Molecular Biology Laboratory, Meyerhofstr. 1, Heidelberg, 69117 Germany; 11 Directors’ Research European Molecular Biology Laboratory, Meyerhofstr. 1, Heidelberg, 69117 Germany; 12 Sorbonne Universités, UPMC Université Paris 06, CNRS, Laboratoire d’oceanographie de Villefranche (LOV), Observatoire Océanologique, Villefranche-sur-Mer, 06230 France; 13 Department of Oceanography, University of Hawaii, Honolulu, 96844 Hawaii USA; 14 Departments of Microbiology and Civil, Environmental and Geodetic Engineering, Ohio State University, Columbus, OH 43210 USA; 15 Department of Biology, Institute of Microbiology, Vladimir-Prelog-Weg 4, Zürich, 8093 Switzerland; 16 Molecular Medicine Partnership Unit, University of Heidelberg and European Molecular Biology Laboratory, Heidelberg, 69120 Germany; 17 Max Delbrück Centre for Molecular Medicine, Berlin, 13125 Germany; 18 Department of Bioinformatics, University of Wuerzburg, Würzburg, 97074 Germany; 19 Aix Marseille Univ, Université de Toulon, CNRS, IRD, MIO, Marseille, 13284 France; 20 Stazione Zoologica Anton Dohrn, Villa Comunale, Naples, 80121 Italy; 21 Cellular and Molecular Microbiology, Faculté des Sciences, Université Libre, de Bruxelles (ULB), Belgium; 22 Interuniversity Institute for Bioinformatics in Brussels, ULB-VUB, Boulevard du Triomphe CP 263, 1050 Brussels Belgium; 23 Department of Marine Biology and Oceanography, Institut de Ciències del Mar (CSIC), E-08003 Barcelona, Catalonia Spain; 24 School of Marine Sciences, University of Maine, Orono, ME 04469 USA; 25 Department of Earth, Atmospheric and Planetary Sciences, Massachusetts Institute of Technology, Cambridge, 02138 MA USA; 26 CNRS UMR 7232, BIOM, Avenue du Fontaulé, Banyuls-sur-Mer, 66650 France; 27 Sorbonne Universités Paris 06, OOB UPMC, Avenue du Fontaulé, Banyuls-sur-Mer, 66650 France; 28 Cell Biology and Biophysics, European Molecular Biology Laboratory, Meyerhofstrasse 1, Heidelberg, 69117 Germany; 29 MARUM, Center for Marine Environmental Sciences, University of Bremen, Bremen, 28359 Germany; 30 PANGAEA, Data Publisher for Earth and Environmental Science, University of Bremen, Bremen, 28359 Germany; 31 Earth Institute, University College Dublin, Belfield, Dublin 4, Ireland; 32 CNRS, UMR 7009 Biodev, Observatoire Océanologique, Villefranche-sur-mer, F-06230 France; 33 National Science Foundation, Arlington, VA 22230 USA; 34 Laboratoire de Physique des Océans, UBO-IUEM, Place Copernic, Plouzané, 29820 France; 35 Department of Geosciences, Laboratoire de Météorologie Dynamique (LMD), Ecole Normale Supérieure, 24 rue Lhomond, Paris Cedex 05, 75231 France; 36 DVIP Consulting, Sèvres, 92310 France

## Abstract

While our knowledge about the roles of microbes and viruses in the ocean has increased tremendously due to recent advances in genomics and metagenomics, research on marine microbial eukaryotes and zooplankton has benefited much less from these new technologies because of their larger genomes, their enormous diversity, and largely unexplored physiologies. Here, we use a metatranscriptomics approach to capture expressed genes in open ocean *Tara* Oceans stations across four organismal size fractions. The individual sequence reads cluster into 116 million unigenes representing the largest reference collection of eukaryotic transcripts from any single biome. The catalog is used to unveil functions expressed by eukaryotic marine plankton, and to assess their functional biogeography. Almost half of the sequences have no similarity with known proteins, and a great number belong to new gene families with a restricted distribution in the ocean. Overall, the resource provides the foundations for exploring the roles of marine eukaryotes in ocean ecology and biogeochemistry.

## Abstract (web summary)

Marine microbial eukaryotes and zooplankton display enormous diversity and largely unexplored physiologies. Here, the authors use metatranscriptomics to analyze four organismal size fractions from open-ocean stations, providing the largest reference collection of eukaryotic transcripts from any single biome.

## Introduction

Single-celled microeukaryotes and small multicellular zooplankton account for most of the planktonic biomass in the world’s ocean^1,2^. They are involved in various processes that shape the biogeochemical cycles of the planet, from primary production, recycling of organic matter by predation and parasitism, sequestration of carbon to a depth, and the transfer of organic material to higher trophic levels in the food webs^3^. Yet, their analysis is confounded because they are represented by hundreds of thousands of different taxa belonging to almost all phylogenetic groups of eukaryotes^4^, and the vast majority of them cannot be cultured. Their highly variable genome sizes, spanning at least four orders of magnitude^5^, and the predominance of noncoding sequences are additional challenges that have impeded their genomic exploration. Consequently, their study has been limited principally to morphological description of diversity, as well as taxonomic and biogeographic characterizations using individual barcode genes^6,7^. By contrast, global surveys of the functional potential of marine microbiota (≤3 µm) and double-stranded DNA viruses are advancing rapidly because of the availability of comprehensive gene catalogs^8–12^, as has been performed for the human gut^13^. To help assess gene function in marine eukaryotes, transcriptome data sets from hundreds of cultured marine eukaryotes^14^ have been generated, as well as from some species of zooplankton^15^, which is helping to analyze features of the global eukaryotic proteome and to interpret the transcriptional responses of some components of eukaryotic communities to localized stimuli^16,17^.

Herein, we use a metatranscriptomics approach using samples collected from the global ocean during the *Tara* Oceans expedition^18^ to generate a global ocean reference catalog of genes from planktonic eukaryotes and to explore their expression patterns with respect to biogeography and environmental conditions.

## Results

### The *Tara* Oceans catalog of expressed eukaryotic genes

**Fig. 1:**
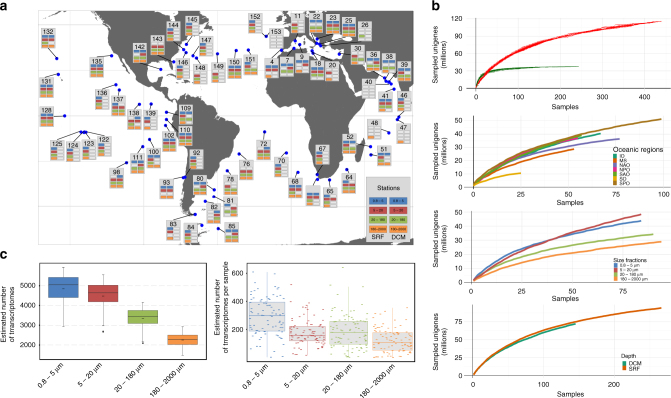
The *Tara* Oceans eukaryote gene catalog. **a** Sampling map. Geographic distribution of 68 sampling stations at which seawater from the surface (SRF) and/or the deep chlorophyll maximum (DCM) was collected and size fractionated into four main groups: 0.8–5 µm (blue), 5–20 µm (red), 20–180 µm (green), and 180–2000 µm (orange). Availability of sequence data sets is indicated by the colored boxes at each sampling station. Two stations (TARA_40 and TARA_153) containing only atypical size fractions are shown on this map with empty boxes. **b** Rarefaction curves of detected genes. Top panel: rarefaction curves of 441 eukaryotic samples (red curve) compared to 139 prokaryotic samples (green curve) derived from Sunagawa et al^9^. Other panels: rarefaction curve of eukaryotic samples by oceanic region (IO, Indian Ocean; MS, Mediterranean Sea; NAO, North Atlantic Ocean; NPO, North Pacific Ocean; SAO, South Atlantic Ocean; SO, Southern Ocean; SPO, South Pacific Ocean), size fraction, and depth (SRF or DCM). For each curve, sampling order has been 10-fold permuted. **c** Estimated number of transcriptomes in eukaryotic samples. Left panel: distribution of the total number of transcriptomes estimated for each size fraction computed from the number of unigenes similar to a catalog of 24 single-copy ribosomal proteins. Right panel: distribution of the number of transcriptomes in each sample (small dashes) grouped by size fraction

To identify and characterize the transcriptionally active genes from the most abundant eukaryotic plankton in the global ocean, we selected samples collected during the *Tara* Oceans expedition at two main depths in the euphotic zone (subsurface (SRF) and deep chlorophyll maximum (DCM)), at 68 different geographic locations across all the major oceanic provinces except the Arctic^19^ (Fig. 1a). Four main organismal size fractions were sampled independently^20^ to optimize the recovery of comprehensive metatranscriptomes from piconanoplanktonic, nanoplanktonic, microplanktonic, and mesoplanktonic communities, covering protists to zooplankton and fish larvae. High-coverage polyA-based (to avoid ribosomal, organellar, and bacterial RNA) RNA-Seq was performed on a total of 441 size-fractionated plankton communities (Fig. 1a), resulting in 16.5 terabases of raw data from which residual ribosomal RNA sequences were removed. The cDNA reads were individually assembled for each sample and then clusterized together at 95% sequence identity to create a single, largely nonredundant resource of 116.8 million transcribed sequences of at least 150 bases in length, hereafter termed unigenes, with a N50 length of 635 bases. Rarefaction analysis revealed that, despite its magnitude, the sampling effort did not result in near saturation of the eukaryotic gene space, contrasting with the results obtained from the smallest prokaryote-enriched size fractions, analyzed by metagenomics from 243 *Tara* Oceans samples^9^ (Fig. 1b). We estimate that the unigene curve would reach saturation at 166–190 million sequences, if all ocean regions would be taxonomically homogeneous (Supplementary Data 1).

**Fig. 2:**
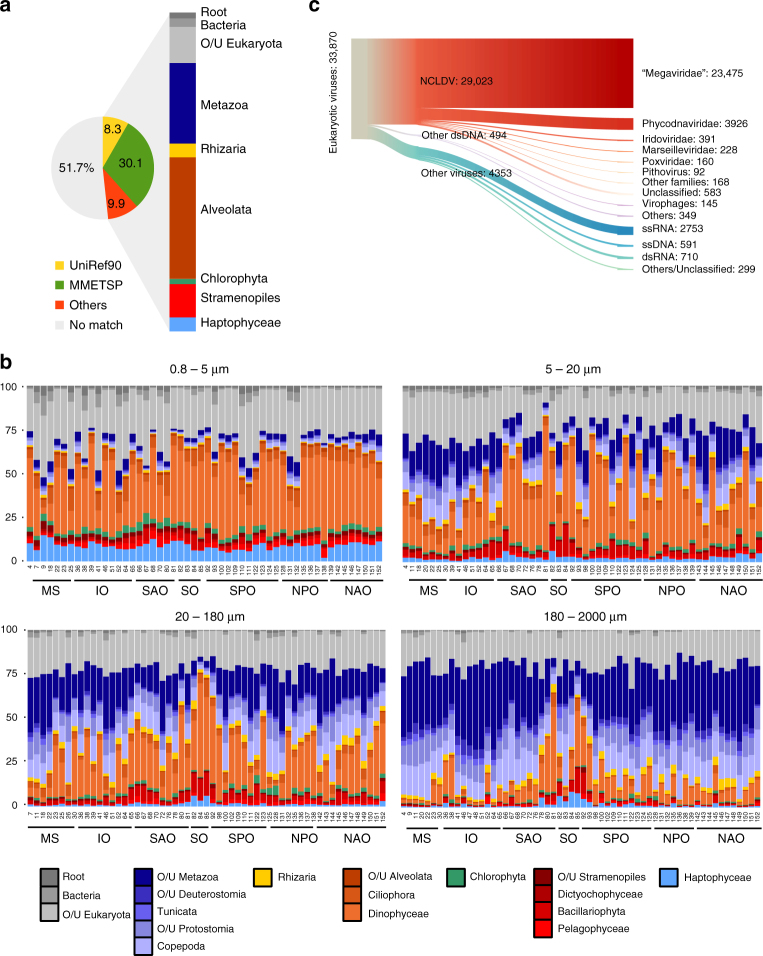
Taxonomic composition of the gene catalog. **a** Origin of the best similarity sequence match as a fraction of the total in the circular diagram (MMETSP^14^: release of August 2014, with manual curation; UniRef90^42^: release of September 2014; “Others”: are other reference transcriptomes that were added as reference to offset the lack of knowledge about organisms in large size fractions, in particular copepods and rhizaria; Methods section). Unigenes without significant matches (i.e., those with an *e*-value >10^–5^ for their best similarity match) are tagged as “No match”. The proportion of unigenes affiliated to each major taxonomic group is indicated in the right column. O/U, other or unassigned. **b** Proportion of each major taxonomic group across *Tara* Oceans stations based on the mean number of unigenes classified as one of 24 different single-copy ribosomal proteins detected in each sample (IO, Indian Ocean; MS, Mediterranean Sea; NAO, North Atlantic Ocean; NPO, North Pacific Ocean; SAO, South Atlantic Ocean; SO, Southern Ocean; SPO, South Pacific Ocean). **c** Eukaryotic viral unigenes. NCLDV unigenes are classified at the family level

Annotation of the >116 million unigenes (Methods and Supplementary Fig. 1a) revealed that we could assign a taxonomy level (from “cellular organism” to species name) to only 48.3% of the unigenes (Fig. 2a and Supplementary Fig. 1b). By mapping the unigenes onto known gene annotations from marine genomes, we found a mean value of 2.20 (s.d. = 0.47) unigenes per gene (Methods and Supplementary Data 2). We then estimated the number of distinct transcriptomes (originating from different species) that were present in the catalog by counting the mean number of copies of conserved ribosomal protein genes, which indicated that the catalog contains genes from 8823 (s.d. = 1532) different organisms (Supplementary Data 3). These values indicate that the unigenes are derived from around 53 (44–68) million genes, with a mean of 6014 (4226–9223) genes per sampled organism (Supplementary Data 4). All sequencing reads from the 441 samples, as well as the reads from a parallel metagenomics sequencing program, were mapped onto the unigenes to provide relative expression and abundance for each gene in every sample (Methods and Supplementary Fig. 1a).

With an equivalent sequencing effort, the complexity of the metatranscriptomes decreased from the smallest piconano-planktonic communities to the largest, mesoplanktonic assemblages (Fig. 1c), matching the pattern observed in extensive rDNA metabarcoding data sets^6^. Rarefaction curves calculated individually per size fraction revealed the higher complexity of the piconano and nanoplankton communities (Fig. 1b), and we found that the 5–20 µm size fraction was the most gene rich, due to intersample dissimilarity and the presence of more gene-rich transcriptomes (Fig. 1b, c). All size fractions contained a significant number of genes not found in the others (8.7–29%; Supplementary Fig. 1c), indicating the importance of size fractionation to describe the global eukaryote gene content of the ocean. With the limitation that we are considering the most expressed genes in our samples rather than the total gene content, we observed that a breakdown of the rarefaction curve by oceanic provinces shows consistent richness and undersaturation of the gene space, with the notable exception of the Southern Ocean, and to a lesser extent of the Mediterranean Sea (Fig. 1b). A high-taxonomic level breakdown of the assignable unigenes across *Tara* Oceans stations and organismal size fractions shows a higher relative abundance of genes from photosynthetic protists in the piconano plankton, and their progressive replacement by metazoan transcripts in larger size fractions (Fig. 2b), confirming the efficiency of the fractionation-based approach. We observed 1.13% of unigenes that are affiliated to prokaryotes. These were not removed from the catalog, as they can be true nonpolyadenylated transcripts from this group, or alternatively to the low level of eukaryotic annotations with respect to prokaryotes in reference databases, or to horizontal gene transfers.

Our metatranscriptomic data also captured transcripts (or RNA genomes) of viruses actively infecting their eukaryotic hosts. Their activities were found to be pervasive across the geographic and organismal size ranges examined in this study. Of the taxonomically assignable unigenes, 33,870 (0.06%) were predicted to be of eukaryotic virus origin, the vast majority of which (86%) originated from nucleocytoplasmic large dsDNA viruses (NCLDVs)^21^ (Fig. 2c) likely due to the large number of genes encoded in these viruses. Eukaryotic viral unigenes were expressed (or present in the case of RNA viruses) in all 441 samples at a relative abundance ranging from 0.0006 to 0.4% (0.02% on average). NCLDV transcripts dominated the piconano-planktonic communities, while RNA virus sequences became dominant with increasing organism size (Supplementary Fig. 2).

### Factors discriminating the most expressed functional classes

To investigate the functional structuring within eukaryotic plankton communities, we defined the main parameters discriminating the Pfam domain profiles using principal component analysis (PCA). The first two axes of the PCA are shown in Supplementary Fig. 3a. The main parameter explaining variance corresponded to differentiation between small-size and large-size fractions (horizontal axis), and the second major component of variance (vertical axis) separated the Southern Ocean (SO) samples from all the others. A few Gene Ontology (GO) terms show consistent patterns across all size fractions, highlighting major functional and taxonomical differences between SO regions and temperate or tropical oceans (Supplementary Fig. 3b), that can be either due to geographic segregation or to specific parameters of SO, e.g., low iron bioavailability. Samples from this region also tend to be more enriched in diatoms than at the other stations (mean 13%, s.d. = 3.8 in austral stations vs. 3%, s.d. = 2.2, in other samples) (Fig. 2b).

**Fig. 3:**
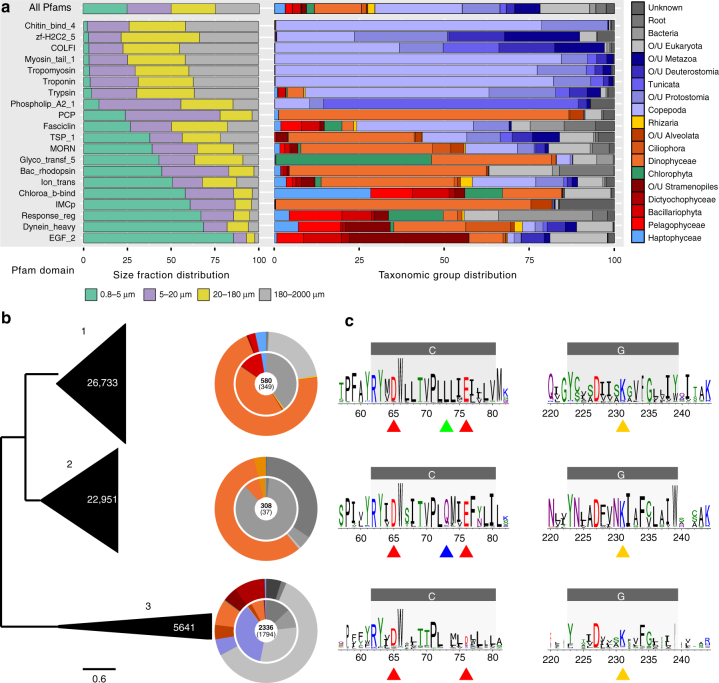
Characterization of highly expressed gene families. **a** Major Pfam domains present in different size fractions and in different taxonomic groups. Among the highly expressed Pfam domains (Supplementary Fig. 4), those with specific patterns are shown. The relative expression of Pfam domains in the four filter sizes (left panel) and the contribution of each taxonomic group to the total expression of the Pfam domain (right panel) are shown as an average of all *Tara* Oceans SRF and DCM samples. O/U, other or unassigned. **b** Unrooted phylogenetic tree of type-I rhodopsin subfamilies (PF01036) obtained using sampling of 300 sequences of the three largest MCL clusters (see details in Supplementary Fig. 5b). The vertical size of the triangles represents the number of unigenes in each cluster (explicitly indicated in white) and their width represents the maximum branch length of 95% of sequences in the cluster. Taxonomic assignments of reference sequences (inner ring) and unigenes (outer ring) are indicated for each cluster with the color code of **a**. The number of reference sequences in each cluster is indicated in the center in bold, with the number of eukaryotic sequences in parentheses. **c** Logo consensus sequences, based on the global alignment of each cluster. Two regions of interest (helices C and G and their neighborhoods) containing functional and conserved residues are represented^25^. Specific functional residues are indicated with arrows. Red: proton donor (D65) and acceptor (E76); green: residue specific to green light-sensitive proteorhodopsins; blue: amino acid specific to blue light-sensitive proteorhodopsins; yellow: lysine residue linked to retinal. Predicted transmembrane helices are represented as gray boxes

When looking at the most enriched gene categories between size classes, we observed small fractions being enriched in light-based energetic processes (photosynthesis and proteorhodopsins), transport of nutrients, carbohydrate metabolism, and flagellar movement, whereas large size fractions were associated with functions related to multicellularity, cell–cell contact, chitin metabolism, and muscular movement (Fig. 3a and Supplementary Fig. 4). This result demonstrates that the metatranscriptomics data capture not only the taxonomic differences observed previously^6^ but also the functional repertoires in each size fraction. We also observed that the relative expression of photosynthesis genes (seen through chlorophyll-binding proteins) vs. proteorhodopsins (Bac_rhodopsin Pfam domain corresponding to type-I rhodopsins^22,23^) showed a strong preference for photosynthesis in groups dominated by autotrophs, supporting that rhodopsin is not a major way of using light energy in these groups in natural conditions (Supplementary Fig. 5a). To further investigate the distribution of the expression of the rhodopsins present in the catalog, we isolated all the unigenes bearing a Bac_rhodopsin Pfam domain. We added to the dataset 2112 proteins—mainly from fungi (40%), bacteria (35%), and archaea (18%)—from public databases and 2538 eukaryotic protein sequences from MMETSP^14^. Protein sequences from the 71,576 unigenes carrying the Bac_rhodopsin Pfam domain were aligned and clustered with reference sequences to study their diversity (Methods section). We found that a large majority of annotated eukaryotic unigenes (82% of unigenes with the Bac_rhodopsin motif) were assigned to alveolates (73%), and contain conserved residues for proton-pumping activity, indicating that this group is the main contributor to proteorhodopsin-based light transduction in the open ocean. The three main clusters contain 55,325 unigenes (77%), and correspond to the three main groups observed based on references only^24^ (Fig. 3b). Cluster 1 contains xanthorhodopsin-like proteins with conserved residues implicated in proton pumping (Fig. 3b, c and Supplementary Fig. 5b). The 26,733 unigenes of this cluster are almost exclusively derived from stramenopiles, alveolates, and haptophytes. This taxonomic distribution is consistent with the proposed single horizontal transfer from a bacterium to the common ancestor of the SAR group (Stramenopiles, Alveolates, and Rhizaria) and Haptista^24^. The third cluster contains a large number of eukaryote references and most known sensory rhodopsins, but only 5641 unigenes with diverse taxonomies. Moreover, the proton acceptor residue E76, involved in the proton-pumping function, is not conserved, indicating that Cluster 3 proteins are likely to represent principally sensory rhodopsins (Fig. 3b, c and Supplementary Fig. 5c). Surprisingly, Cluster 2 contains only a few eukaryotic references but is the second largest with 22,951 sequences, and displays the consensus sequence consistent with a proton-pumping function (Fig. 3b, c and Supplementary Fig. 5d). Most of these appear to be derived from alveolates, including the syndiniales parasites. This indicates that one of the most important categories of proteorhodopsins in the ocean is currently underestimated, possibly because of the lack of cultivated organisms bearing it, and that it may link photoheterotrophy with parasitism, a currently unexplored topic. Based on the hypothesis of a single lateral gene transfer event^24^, the restricted taxonomic distribution of unigenes in Cluster 2 suggests a more recent acquisition, which probably occurred before or during the radiation of the alveolate lineage. Interestingly, the consensus spectral tuning residue is different between Cluster 1 and Cluster 2: Cluster 1 protein sequences exhibit a leucine at position 105^25^, indicating a maximal absorption of green light, whereas Cluster 2 sequences bear a glutamic acid at this position, indicating a peak absorption of blue wavelengths (Fig. 3c).

### Gene novelty

**Fig. 4:**
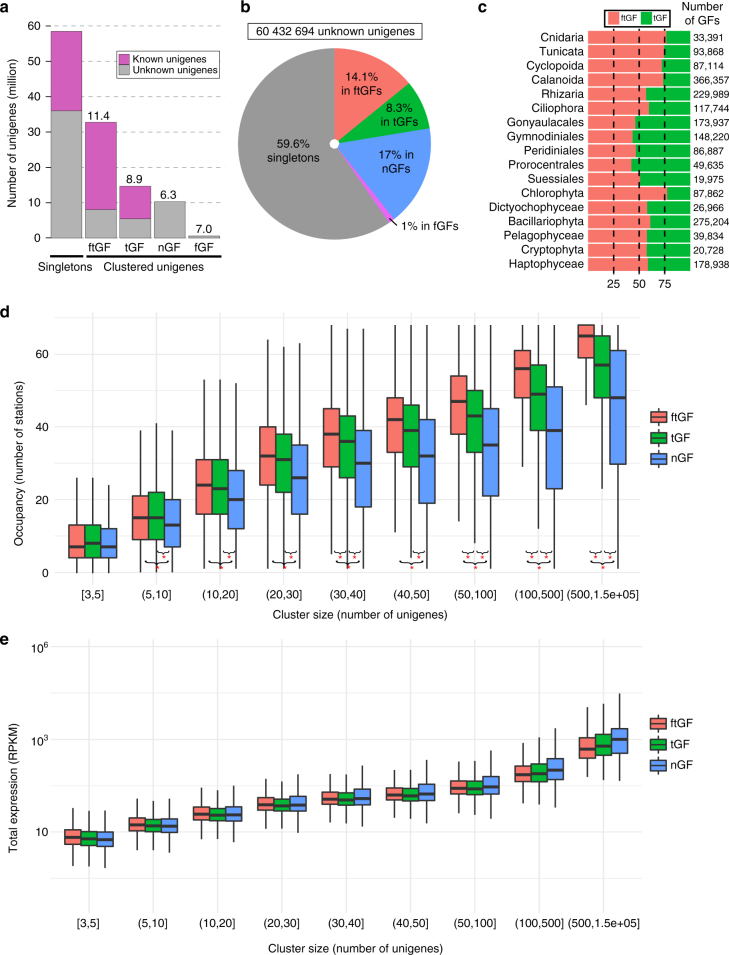
Eukaryote gene catalog clustering and characterization of novel genes. **a** Global repartition of unigenes based on the gene catalog clustering. Unigenes were considered as singletons if they are in clusters of less than three units. Gene families are novel (nGF), taxonomically assigned (tGF), functionally assigned (fGF), or both (ftGF) (Methods). Numbers above each bar indicate the numbers of unigenes per cluster. **b** Distribution of unknown unigenes in the different categories described in **a**. **c** Ratio of tGFs vs. ftGFs in the main taxonomic groups. The total number of GFs assigned to each taxonomic group is indicated on the right. **d** Distribution of GF occupancy for the three main GF categories. GFs are classified according to their size (*x*-axis) and the *y*-axis indicates the number of stations where the GF family is expressed (at least one unigene detected with a coverage of more than 80% of the unigene length). Kolmogorov-Smirnov tests with *p* < 10^–5^ between occupancy distributions are indicated with red stars. **e** Distribution of mean expression levels of the three different categories of GFs among all samples. GFs are classified according to their size (*x*-axis). The expression of a GF in a sample was determined by the sum of the expression of its unigenes in RPKM

The majority (51.2%) of unigenes currently have no matches in public sequence databases, which limits the insights that can be derived from the gene catalog. Some sequences may be derived from non-coding genes or non-coding portions of coding genes, very short open reading frames, parts of genes where only another region is functionally known, or completely new open reading frames. To distinguish between these possibilities and better classify the catalog, we clustered all the unigenes according to a nucleic acid similarity threshold of >70% (Methods; Supplementary Fig. 6a). Despite its size, the gene catalog is not saturated, and accordingly we observed that 59.6% of unknown unigenes (UU) and 39.8% of known unigenes are represented by singletons (Fig. 4a, b). The clusters may thus be considered as being representative of gene family (GF) content of the catalog, with most singletons likely being derived from smaller GFs that will grow with more sequencing effort. The 6.2 million GFs, encompassing 58.4 million unigenes, were subsequently subdivided into four classes based on taxonomic affiliation and functional annotation (see Methods; Fig. 4a-c): those with both functional and taxonomic assignments (ftGF), those with taxonomy-only assignments (tGF), those with function-only assignments (fGF), and those representing new GF (nGF). The fGF category was not considered further because it contains too few clusters (1.43%).

We searched for fundamental differences between these three types of GFs by observing in how many stations they were detected (Methods section). Regardless of GF size, nGFs were present in less stations than ftGFs, whereas tGFs showed intermediate occupancies (Fig. 4d and Supplementary Fig. 7a). This pattern was not due to higher mean expression levels of ftGFs or tGFs that would render them more detectable than nGFs (Fig. 4e). We conclude that the gene novelty detected corresponds to families that are present in fewer environments, yet are not less expressed than known families. Moreover, nGFs generally represent smaller GFs (6.3 unigenes per cluster) than fGFs (8.9) and ftGFs (11.4), suggesting that nGFs are conserved in a smaller range of species than characterized GFs (Fig. 4a and Supplementary Fig. 6b), or that they are present in less abundant taxonomic groups. It has been previously suggested that newly discovered genes are either biased taxonomically (which restrains their presence in databases), or that they correspond to genes that are necessary only in some conditions, potentially related to the adaptation of organisms to specific environments^26^. We found evidence for both cases, as nGFs are more restricted in occupancy, whereas tGFs are more abundant in less-characterized phyla (Fig. 4c-e).

We further questioned whether the intermediate occupancies observed with tGFs can be due to an intrinsic property or to them being distributed between two types of families, looking either more like ftGFs or more like nGFs. The distribution of occupancies in tGFs indeed appears to be bimodal, with a group containing fewer UUs resembling the ftGF distribution, and another group containing a high proportion of UUs resembling the nGF distribution (Supplementary Fig. 7b,c). We conclude that some of the tGFs likely represent widely occurring genes that have no predicted functions, most likely because of their limited taxonomic distribution in the global tree of eukaryotes. The others may represent GFs with characteristics of nGFs that have few members matching with references, generally reflecting efforts to gain information on environmentally-important organisms such as the MMETSP effort^14^.

**Fig. 5:**
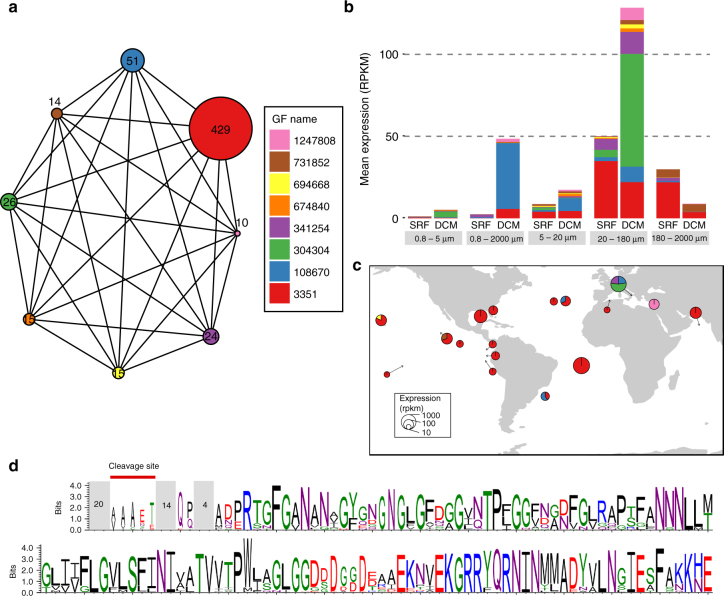
New gene families expressed in 20–180 μm size fraction. **a** Graph representation of the protein group number *14079*. Each GF of the protein group is represented by a node with a diameter proportional to the number of unigenes in the GF. Protein matches between GFs are represented by an edge. **b** Mean expression of GFs in different size-fractions and depths. Each color corresponds to a GF of protein group *14079*. **c** World map representation of protein group *14079* expression in the 20–180 µm size fraction. SRF and DCM samples have been pooled. Circle diameters represent the relative expression of the protein group in RPKM. The contribution to expression of each GF is represented by the different colors. **d** Sequence logo of the multiple alignments of the protein group *14079*. 45 ORFs (153 amino acids in average) of protein group *14079* were aligned and positions with more than 50% of gaps were removed. Mean numbers of amino acids on unaligned regions of the protein are indicated in gray boxes. A signal peptide cleavage site, indicated on the left part of the sequence logo was predicted on 21 sequences

Although our metatranscriptomics sequencing effort is based on polyadenylated RNA and relatively shallow coverage per individual organism, and thus may not be able to capture non-coding RNAs significantly, we then consider the nGF category, asking if these new families can be coding. For this, we selected the central unigene of each cluster of more than 10 unigenes as a reference of the GF, then we looked for protein homologies between references (see Methods and Supplementary Fig. 6a). This created 75,175 protein groups of GFs, among which 11,431 link 30,558 nGFs only, and 22,072 link 130,501 tGFs only. Examples of nGFs are shown in Fig. 5 (protein group number *14079* for nGFs with restricted expression) and Supplementary Fig. 8a–d (protein group number *1540* for more broadly distributed nGFs). We were able to align ORFs from these clusters and found that they contain highly conserved amino acids that can provide clues about their structure (Fig. 5d, Supplementary Fig. 8d). Another example from a highly conserved tGF restricted to dinoflagellates and close relatives is shown in Supplementary Fig. 8e–h. Taken together, these data show that 3.26 million GFs with or without taxonomic information are present as highly expressed families in the global ocean and do not correspond to defined domains. We suggest that these may be important targets for future definition of new protein domains to more faithfully encompass the functional diversity present in eukaryotes. The current database of protein domains such as Pfam^27^ contains 16,712 different domains of known and unknown functions, whereas we detected 11,431 protein groups of nGFs, and 22,072 groups of tGFs based only on Clustering of the largest families, indicating the high discovery rate of new conserved domains that could be used to derive a more exhaustive list of conserved domains within eukaryotes.

In summary, we have found that UUs can be part of known GFs but that a large proportion are predicted to be novel protein-coding genes. As they are distributed less globally than known functions, their extent remains to be evaluated, although we have shown here that they represent a highly significant portion of the gene repertoire of eukaryotic plankton.

### The environmental footprint of gene expression in phytoplankton

To highlight how the annotated gene catalog can be useful for studying environmental gene expression, we examined the five principal photosynthetic groups (Fig. 2c), namely diatoms (Bacillariophyta), chlorophytes, dinoflagellates (Dinophyceae), haptophytes, and pelagophytes, for some of their most highly expressed functions and their variations according to two environmental parameters, specifically iron and net primary production (NPP). Obligate autotrophs, such as diatoms and chlorophytes, showed a higher correlation to NPP for genes involved in photosynthesis and carbon fixation than the other groups that also contain mixotrophic representatives. Additionally, we observed an apparent lack of correlation between expression of genes important for photosynthesis and carbon fixation in dinoflagellates in conditions of high NPP (Supplementary Fig. 9). Although this could be explained by low reliance on transcriptional regulation in this group^5^, we observed an increased correlation of expression of genes encoding cell lytic components, such as proteases and lipases. Such changes in ecosystem function may be a consequence of alterations in the dominant dinoflagellates in the community or to switches in trophic strategy in mixotrophic species, and have significant implications for the functioning of marine food chains in different environmental conditions.

Differences in expression patterns of unigenes between two sampling stations can be linked to either (or both) changes in population composition and changes in expressed functions related to the environment. Comparison of metagenomes and metatranscriptomes allows assessment of the expression of genes from the catalog normalized to underlying gene abundances. To highlight this, we examined genes whose expression and/or copy number have been shown to be responsive to nutrient availability, specifically iron, an important yet often limiting nutrient in the ocean.

**Fig. 6:**
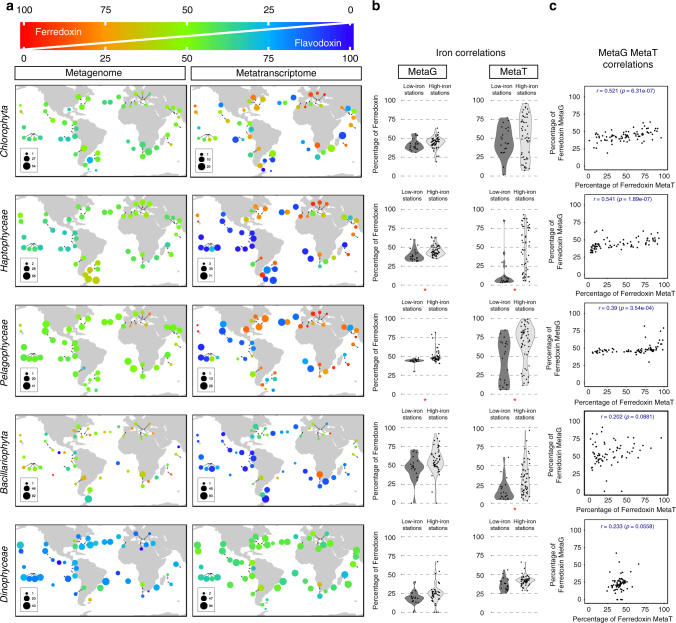
Ratios of differential gene abundance and relative expression of ferredoxin vs. flavodoxin in the five major photosynthetic groups. **a** Representation of the relative abundance (left) and expression (right) of the two genes identified in surface samples for *Chlorophyta*, *Pelagophyceae*, *Haptophyceae* (from 0.8 to 5 µm filters), *Bacillariophyta* and *Dinophyceae* (from the 5 to 20 µm filters). The circle colors, from red to blue, represent the relative expression of one gene compared to the other, with the color code given in the top diagram. The sum of the expression levels of the two genes affiliated to each taxonomic group is represented by the circle diameter as a percentage of the total expression of these genes. **b** Distribution of the relative abundance (left) or expression (right) of ferredoxin in low iron stations (<0.02 µmol m^−3^, 15 stations, dark gray) or iron rich stations (>0.2 µmol m^−3^, 31 stations, light gray) according to a model of iron concentration in the oceans (Supplementary Data 5). Significant differences of expression between low and rich iron stations are indicated with red stars (non-parametric wilcoxon rank-sum test, *p* < 10^–3^) **c** Correlations between the relative metagenome (MetaG) abundance and metatranscriptome (MetaT) expression of ferredoxin in SRF and DCM samples, expressed as a percentage of the total value of ferredoxin + flavodoxin. Pearson correlation coefficients (*r*) and their statistical significance (*p*) are indicated in each graph. Ferredoxins and flavodoxins were identified using the Pfams PF00111 and PF00258, respectively

Phytoplankton are good models to study iron homeostasis as they have significant high demands of this metal due to its requirement for photosynthesis^28^. One low iron response that occurs in the photosynthetic electron transport chain involves the replacement of the iron-sulfur containing electron carrier ferredoxin with flavodoxin, a less efficient protein that does not require iron^29,30^. In addition to the canonical photosynthetic versions, there are a number of flavodoxins and ferredoxins involved in different metabolisms, or constituting functional domains of complex multidomain redox proteins^29^. To study whether the flavodoxin/ferredoxin switch can be detected using our dataset, we carried out an analysis of the ferredoxin and flavodoxin families using the Pfam domains PF00111 and PF00258. These families not only include the photosynthetic versions but also other isoforms and domains, and there is an overlap of redox properties between different members of these two families, being potential isofunctional proteins in many reactions^29^. Thus, we studied the relative levels of the two families of genes in the five major phytoplankton groups by calculating the ratio of their relative abundances and expression (Fig. 6). With the exception of diatoms, gene abundances show little variations and only weak correlations with iron concentrations (Fig. 6a; “Metagenome” column and Supplementary Data 5). On the other hand, the ratios of relative expression show strong variations, particularly for chlorophytes, haptophytes and pelagophytes (Fig. 6; “Metatranscriptome” column), indicating that these three groups modulate the relative levels of ferredoxin and flavodoxin principally by regulation of mRNA levels. By contrast, diatoms tend to express flavodoxin genes more than ferredoxin genes, although a few mainly coastal stations showed a strong up-regulation of the latter. In this group, the metagenomics data indicate that diatom genomes display far more heterogeneity in ferredoxin/flavodoxin content than the other groups studied, suggesting that individual diatom species may be permanently adapted to specific iron regimes in the ocean rather than maintaining transcriptional flexibility, as observed in haptophytes, chlorophytes and pelagophytes. Unlike any other groups, dinoflagellates appear to rely only weakly on gene abundance or expression variations (Fig. 6), which may again be related to their low transcription flexibility. These results suggest that nutrient limitations are dealt with in different ways among these main photosynthetic taxa, either by a genotypic commitment to a specific regime, or by the maintenance of transcriptional flexibility, and that the *Tara* Oceans eukaryote gene catalog may be a useful resource to distinguish the strategies of any plankton group to adapt to these limitations when transcript regulation or gene copy number is implicated.

## Discussion

The global ocean transcript catalog reported here represents a first resource to study extensively and uniformly the gene content of eukaryotes and the dynamics of their expression in the environment, and notably adds to previous DNA-based resources that describe the viral and prokaryotic components of the ocean^9–11^. The gene repertoire of planktonic eukaryotes is massive and diverse, much more so than the prokaryotic gene space^9^. The impressive number of genes without functionally-characterized homologs in databases points to the large numbers of understudied yet widely distributed genera inhabiting marine ecosystems, for which even widely conserved GFs have yet to be investigated. The restricted distribution of totally new GFs highlights the need to develop methods for revealing their roles without the support of homology-based hypotheses. Because representatives of almost all of the eukaryote groups^4^ are abundant in oceanic plankton, they can likely inform us in new ways about the evolutionary trajectories of different eukaryotes, in particular those with parasitic and symbiotic lifestyles that have remained largely recalcitrant to study until now, although being a large part of the interacting species network within plankton ecosystems^31,32^. The resource is also likely to be of great utility for exploring organisms within the zooplankton, including metazoans, that have to date been largely unexplored by genomics^33^. As we have shown for the principal groups of phytoplankton, it is possible to obtain insights between adaptive and acclimatory processes underlying organismal responses to their environment using as proxies the contrasts between metagenomics and metatranscriptomics, paving the way for similar studies in other organisms.

## Methods

### Sampling of eukaryotic plankton communities

The biological samples were collected during the *Tara* Oceans expedition from 68 sampling sites. Typically, two depths were sampled in the photic zone: subsurface (SRF) and deep-chlorophyll maximum (DCM). A detailed description of all *Tara* Oceans field sampling strategy and protocols is available in Pesant et al^20^. In short, planktonic eukaryote communities were collected in the 0.8–2000 µm range and size-fractionated in four fractions (0.8–5 μm, 5–20 μm, 20–180 μm, and 180–2000 μm). A low-shear and non-intrusive industrial peristaltic pump was used for the 0.8–5 µm fraction and plankton nets for the others. The volumes of filtered seawater were scaled according to known organismal concentrations within each size fraction, from 0.1 m^3^ for the most concentrated pico-plankton to 148 ± 136 m^3^ for the most-dilute meso-plankton, in order to get near-exhaustive recovery of total eukaryotic biodiversity in each sample. Water was filtered immediately after sampling. Whole-plankton communities were subsequently filtered on polycarbonate membranes, rapidly flash-frozen and preserved in liquid nitrogen on board *Tara*.

Physicochemical parameters measured during the expedition are available in the Pangaea database (https://www.pangaea.de/ and Supplementary Data 5) and described in Pesant et al^20^. Due to the sparse availability of direct observations of iron in the surface ocean, concentrations were derived from a global ocean simulation using the MITgcm ocean model configured with 18 km horizontal resolution and a biogeochemical simulation which resolves the cycles of nitrogen, phosphorus, iron and silicon^34^. The biogeochemical parameterizations, including iron, are detailed in Follows et al^35^. Atmospheric deposition of iron was imposed using monthly fluxes from the model of Mahowald et al^36^. NPP values were derived from satellite measurements from 8-day composites of the vertically generalized production model^37^. Physicochemical parameters of each station analyzed in this article are indicated in Supplementary Data 5.

### Nucleic acid extraction, library construction, and sequencing

DNA and RNA were extracted simultaneously by cryogenic grinding of cryopreserved membrane filters using a 6770 Freezer/Mill or 6870 Freezer/Mill instrument (SPEX SamplePrep, Metuchen, NJ) followed by nucleic acid extraction with NucleoSpin RNA Midi kits (Macherey-Nagel, Düren, Germany) combined with DNA Elution buffer kit (Macherey-Nagel). DNA and RNA were quantified by a fluorometric method using Qubit 2.0 Fluorometer (ThermoFisher Scientific, Waltham, MA). DNase treatments were applied to all RNA extractions. Metagenomic libraries were prepared manually or in a semi-automatic manner according to available DNA quantity. Genomic DNA was first sheared to a mean target size of 300 bp using a Covaris E210 instrument (Covaris, Woburn, MA). DNA inputs in fragmentation step were 30–100 ng in the case of a downstream manual preparation or 250 ng for semi-automatized protocol. End repair, A-tailing and Illumina adapter ligation were then performed manually using NEBNext Sample Reagent Set (New England Biolabs) or with the SPRIWorks Library Preparation System and SPRI TE instrument (Beckmann Coulter Genomics), according to the manufacturers protocol. Ligation products were PCR-amplified using Illumina adapter-specific primers and Platinum Pfx DNA polymerase (Invitrogen). Amplified library fragments were size selected at around 300 bp on 3% agarose gels. For RNA samples, a poly(A)^+^ RNA selection strategy was used to limit rRNA quantity. Different cDNA synthesis protocols were applied according to the quantity of RNA. When at least 2 µg total RNA were available, cDNA synthesis was carried out using the TruSeq mRNA Sample preparation kit (Illumina, San Diego, CA). Samples with less than 2 µg of RNA were processed using the SMARTer Ultra Low RNA Kit (Clontech, Mountain View, CA). In these cases, fifty nanograms or less of total RNA were used for cDNA synthesis, followed by 12 cycles of PCR preamplification of cDNA and Covaris shearing to a 150–600 bp size range. cDNAs were then used for Illumina library preparation following the manual protocol described for metagenomic libraries, except that the size selection step on agarose gel was omitted. A detailed description of nucleic acid extractions and library construction protocols is available in Alberti et al^38^. After library profile analysis by Agilent 2100 Bioanalyzer (Agilent Technologies, USA) and qPCR quantification (MxPro, Agilent Technologies, USA), libraries were sequenced on HiSeq2000 instruments (Illumina) with a read length of 101 bp in a paired-end mode. In average, 160 million reads per sample were obtained.

### Reads, assembly and gene catalog construction

An Illumina filter was applied to remove the least reliable data from the analysis. The raw data were filtered to remove any clusters with too much intensity corresponding to bases other than the called base. Adapters and primers were removed on the whole read and low quality nucleotides were trimmed from both ends (while quality value is lower than 20). Sequences between the second unknown nucleotide (N) and the end of the read were also removed, as were reads with a resulting length smaller than 30 bp, as well as their mates mapped onto run quality control sequences (PhiX genome). After cleaning, all single reads (fragment with one discarded read) were eliminated from further analyses. Ribosomal RNA-like reads were excluded using sortmeRNA^39^. Resulting reads from each metatranscriptomic sample were assembled using velvet v.1.2.07^40^ with a kmer size of 63. Isoform detection was performed using oases 0.2.08^41^. Contigs smaller than 150 bp were removed from further analysis. Assembly results and descriptive statistics for each sample are shown in Supplementary Data 6. Similar sequences from more than one sample were removed using Cdhit-est v 4.6.1, with the following parameters: -id 95 -aS 90 (95% of nucleic identity over 90% of the length of the smallest sequence). For each cluster of contigs, the longest sequence was kept as reference for the gene catalog. Ribosomal, chloroplastic, and mitochondrial sequences were removed from the resource after blast comparisons and Pfam domains identification. Prokaryote 16S-like unigenes were mega-BLAST scanned for removal. Mitochondrial or chloroplastic sequences were removed based either on the basis of a positive BLAST hit against dedicated reference databases manually curated, and having matches with at least 70% identity over at least 80% of the unigene length or at least 300 bp long, or based on the presence of specific protein domains identified by CDD search. Domains COX1, COX2, COX3, COX2_TM, Cytochrom_B_N_2, Cytochrom_B_C, Cytochrom_B_N, Oxidored_q1, Oxidored_q2, Oxidored_q3, Oxidored_q4, Oxidored_q5_N, Oxidored_q1_N, NADHdh, NDH_I_M, NDH_I_L, and ATP_synt_6_or_A were used as signature for mitochondrial based genes, domains and Photo_RC, PsaA_PsaB, PSII, RuBisCO_large, and RuBisCO_large_N for the chloroplastic ones, while unigenes also bearing domains Peptidase_M41, Gp_dh_N, or Gp_dh_C, GAPDH-I were kept in the resource, being considered as nuclear genes. In summary a unigene as defined here is a complete or partial transcript assemble from metatranscriptomic reads of at least one *Tara* Oceans station. The gene catalog is accessible at http://www.genoscope.cns.fr/tara/.

### Taxonomic assignment

To assign a taxonomic group to each unigene, a reference database was built from UniRef90 (release of 2014–09–04)^42^, from the MMETSP project (release of 2014–07–30)^14^ manually curated to remove sequence redundancy, from *Tara* Oceans Single-cell Amplified Genomes (PRJEB6603)).The database was supplemented with three Rhizaria transcriptomes (Collozoum, Phaeodaea and Eucyrtidium, available through the European Nucleotide Archive under the reference PRJEB21821 (https://www.ebi.ac.uk/ena/data/view/PRJEB21821) and transcriptomes of *Oithona nana*^33^. Sequence similarities between the gene catalog and the reference database were computed in protein space using Diamond (version 0.7.9)^43^ with the following parameters: -e 1e-5 -k 500 -a 8. Taxonomic affiliation was performed using a weighted Lowest Common Ancestor approach. For each unigene, all protein matches with a bitscore value ≥90% of the best match bitscore were kept. For each taxon, only matches with the highest bitscores were retained, and total LCA and weighted LCA (covering at least 67% of all bitscores), were further computed. In order to limit the number of false taxonomic assignments explained by the lack of reference genomes, the LCA result was corrected according to the percentage of identity of selected matches. The maximal taxonomic precision allowed was corrected as follows: >95% of identity = species, <95% of identity = genus, <80% of identity = family, <65% of identity = order, <50% of identity = class. The taxonomic assignment of unigenes is accessible at http://www.genoscope.cns.fr/tara/. The taxonomic assignment of eukaryotic viruses was performed as explained above but with the following modifications. First, all subject sequences with viral taxonomic identifiers were removed and replaced by viral sequences of Virus-Host DB^44^ (as of 23 February 2017) to allow access to host type information. Viral unigene sequences assigned to bacteriophages or archaeal viruses were discarded from analysis. Second, we used the NCLDV nomenclature derived from the common ancestor hypothesis^45^ based on seven distantly related viral families: ‘Megaviridae’, *Phycodnaviridae*, *Marseilleviridae*, *Iridoviridae*, *Ascoviridae*, *Asfarviridae,* and *Poxviridae*. Among these, “Megaviridae” is a recently proposed family^46,47^. We added the following viral groups: *Pandoravirus*, *Pithovirus*, *Mollivirus* proposed to form new NCLDV families^48^ as well as *Faustovirus*^49^. Unclassified virophages were classified as “dsDNA viruses, no RNA stage”. Virophages *Mavirus* and Organic Lake virophages were classified as unclassified virophages. RNA viruses reported in^50^ were classified in their respective order or family according to their phylogenetic position. Viral groups were added for the newly described families Chuviridae^51^, Yanvirus, Weivirus, Zhaovirus, Qinvirus, and Yuevirus^50^. Finally, the LCA result was corrected according to the percentage of identity of selected matches as follows: >95% of identity = species, < 95% of identity = genus, <70% of identity = family.

### Functional characterization of unigenes

Protein domain prediction was performed using the hmmsearch tool of the the HMMer package (version 3.1b2)^52^ against the Pfam-A database (release 28). Only matches exceeding the internal gathering threshold (–cut_ga) were retained. Pfams often detected on the same unigenes were grouped together in a single name (i.e., Arrestin_C;Arrestin_N). These associations of Pfams followed two criteria: (1) The number of unigenes carrying the two pfams is higher or equal to 30% of the average number of unigenes carrying each Pfam. (2) The number of unigenes carrying the two pfams was higher than 30. The list of associated Pfams is given in Supplementary Data 7. The functional characterization of unigenes is accessible at http://www.genoscope.cns.fr/tara/. Unigenes without Pfam domains are excluded from analyses presented in Figs. 3, 6 and Supplementary Figs. 3, 4, 5, 9. The Pfam domain PF01036 was searched in unigenes and the MMETSP collection using hmmscan (from HMMer 3.1b2)^52^. NCBI sequences carrying the Pfam motif were retrieved through the PFAM portal (http://pfam.xfam.org/, May 2017). All-vs.-all BLAST comparisons were run at the protein level using BLAST + 2.6.0 and sequences were clustered with the MCL algorithm^53^ using the -log(*e*-value) as edge weights and an inflation parameter of 1.4. For each of the three largest clusters, protein sequences were aligned using MAFFT 7.31^54^ and positions with more than 50% of gaps were discarded. Logo consensus sequences were created using weblogo 3 program^55^. Transmembrane helices were predicted using TMHMM Server 2.05 on the consensus sequences^56^. Global phylogenetic tree was constructed from a global alignment using MAFFT 7.310. The phylogenetic inference was made using approximate maximum likelihood with FastTree^57^, under the gamma model of heterogeneity.

### Expression and abundance of unigenes

In order to estimate the abundance and expression of each unigene in each sample, cleaned reads (from metagenomes and metatranscriptomes) were mapped against the reference catalog using the bwa tool (version 0.7.4)^58^. The following parameters were used: bwa aln -l 30 -O 11 -R 1; bwa sampe -a 20000 -n 1 –N; samtools; rmdup. Low complexity reads were removed. Reads covering at least 80% of read length with at least 95% of identity were retained for further analysis. In the case of several possible best matches, a random one was picked. Mapping results are summarized in Supplementary Data 8. Unigene expression values and genomic occurrences were computed in RPKM (reads per kilo base covered per million of mapped reads). RPKM values for each Unigenes in each sample are accessible at http://www.genoscope.cns.fr/tara/. The abundance or expression of each unigene was normalized and formulated in two different ways. (i) The gene expression/abundance relative to the expression/abundance of all genes from the same taxon in percentage. e.g., the expression of Pelagophyceae Ferredoxin genes (Pfam Fer2, 372 unigenes) represents 0.17% of Pelagophyceae transcriptomes. (ii) The fraction of the gene expression/abundance attributed to a particular taxonomic group. e.g., 24.3% of ferredoxin genes are expressed/present in Pelagophyceae transcriptomes. These normalized values of expression and abundance are calculated for all unigenes grouped by Pfams or GO term (Biological Processes) and a list of taxonomic groups: *Haptophyceae*, *Pelagophyceae*, *Bacillariophyta*, *Dictyochophyceae*, O/U *Stramenopiles*, *Chlorophyta*, *Dinophyceae*, *Ciliophora*, O/U *Alveolata*, *Rhizaria*, *Copepoda*, O/U *Protostomia*, *Tunicata*, O/U *Deuterostomia*, O/U *Metazoa*, O/U *Eukaryota*, *Bacteria*, root (unigenes with matches in at least two of the Eukaryota, Archaea, Bacteria, and Virus superkingdom), unknown (unigenes that have no similarities in amino acid databases)), O/U = unigenes for which taxonomic affiliation ended at the indicated level or belonged to minor classes of the affiliation.

### Estimation of transcriptome diversity

A total of 24 ribosomal genes, single copy, highly expressed and universally distributed^59^, were selected to estimate the number of different transcriptomes in each sample: COG0049, COG0052, COG0080, COG0081, COG0087, COG0088, COG0091-COG0094, COG0096-COG0100, COG0102, COG0103, COG0184-COG0197, COG0200, COG0256, COG0522. The average number of unigenes carrying each of these COG domains was used to estimate the number of different transcriptomes. A unigene was considered to be present in a sample if at least 80% of its length was covered by sample reads with at least 95% identity. Reference genomes and their annotation used to estimate the redundancy of the gene catalog and refine transcriptome diversity estimations were downloaded from Ensembl Protists (http://protists.ensembl.org/index.html) for *Emiliania huxleyi*, *Thalassiosira oceanica*, *Aureococcus anophagefferens*, *Acanthamoeba castellanii* str. Neff and *Monosiga brevicollis*, from Orcae (http://bioinformatics.psb.ugent.be/orcae/) for *Bathycoccus prasinos* and *Micromonas pusilla* and from Genoscope (http://www.genoscope cns.fr/externe/GenomeBrowser/) for *Oikopleura dioica* and *Oithona nana*. The gene catalog was aligned (BLAT v32 × 1) against predicted genes from reference genomes with a minimum of 70% of identity over at least 80% of the length of the smallest sequence of the pair (Supplementary Data 2), then fully overlapping unigenes have been removed. For each reference genome, the average number of unigenes mapping each gene and ribosomal proteins listed above were calculated. The mean of the result for each genome was used as an estimation of the catalog redundancy.

### Construction of gene families

Nucleic acid homologies between all unigenes of the eukaryotic gene catalog were calculated with BLAT (v. 36) (min 70% of identity and 100 bp). The 1609 million matches obtained were clustered with MCL (v. 14–137) into 6,225,695 clusters of 3 unigenes or more, named GFs (Supplementary Fig. 6a, steps 1–2). Clusters were classified into four categories according to their percentage of unigenes with a taxonomic affiliation and/or a functional characterization. Functionally and taxonomically assigned GFs (ftGFs) comprise >5% of unigenes with matches and domains; taxonomically assigned GFs (tGFs) comprise >5% of unigenes with matches but no predicted domains; new GFs (nGFs) have <5% of unigenes with matches or domains; and functionally assigned GFs (fGFs) have >5% of unigenes with domains and <5% with matches (Supplementary Fig. 6a, step 3). The most precise taxonomic affiliation carried by more than 50% of known unigenes of a given tGF or ftGF was chosen to determine its taxonomic affiliation. A representative unigene for each GF with a minimum of 10 unigenes was determined by the calculation of the betweenness centrality (library Graph::Undirected, Perl) of the corresponding MCL cluster. 1,261,965 central unigenes were 6-frames translated, and similarities between them were then computed with Diamond (version 0.7.9)^43^. The best match for each sequence pair with an *e*-value < 1e^−10^ was selected, then all protein matches were clustered with MCL (pondered by the cluster size) (Supplementary Fig. 6a, steps 4–5). MCL clusters of GFs are named protein groups. GFs and protein groups composition and annotation are accessible at http://www.genoscope.cns.fr/tara/. Protein groups detailed in Fig. 5 and Supplementary Fig. 8 were analyzed for their amino acid composition. The 5 longest ORFs with a minimum of 150 amino acids found in each GF of the protein group were aligned with mafft (v. 7.310)^54^ in globalpair mode and unalignlevel at 0.9. The alignment was manually curated in order to remove non-relevant ORFs, then positions with more than 50% of gaps were removed. Peptide signal sequences and cleavage sites were detected with signalP^60^ and added to the alignment. the sequence logo representations were made with weblogo program^55^.

All statistical analyses and graphical representations were conducted in R (v 3.1.2) with R packages ggplot2 (v 2.1.0). The PCA results shown in Supplementary Fig. 3 were obtained using the R package FactoMineR v 1.32, world maps with maps (v 3.1), phylogenetic trees with ggtree (v 1.6.11), and graph representation Fig. 5a and Supplementary Fig. 8a,e with igraph (v 1.0.1) and ggnetwork (v 0.5.1).

### Code availability

Computer codes are available from the corresponding authors upon request.

### Data availability

Sequencing data are archived at ENA under the accession number PRJEB4352 for the metagenomics data and PRJEB6609 for the metatranscriptomics data (see Supplementary Data 8 for details). Unigene catalog is available at ENA under accession number ERZ480625. Environmental data are available at PANGAEA (URLs for each sample are indicated in Supplementary Data 5). The Marine Atlas of *Tara* Oceans Unigenes (MATOU) along with functional and taxonomic annotations, unigenes abundances, expression levels and GFs are accessible at http://www.genoscope.cns.fr/tara/. Other relevant data are available in this article and its Supplementary Information files, or from the corresponding authors upon request.

## Electronic supplementary material

Supplementary Information

Description of Additional Supplementary Files

Supplementary Data 1

Supplementary Data 2

Supplementary Data 3

Supplementary Data 4

Supplementary Data 5

Supplementary Data 6

Supplementary Data 7

Supplementary Data 8
