# Supplementary material for: A global ocean atlas of eukaryotic genes
Source: Nat Commun. 2018 Jan 25;9:373. doi: 10.1038/s41467-017-02342-1 (PMC5785536; doi:10.1038/s41467-017-02342-1)
Supplement: Supplementary file 1 — Supplementary Information [file 41467_2017_2342_MOESM1_ESM.pdf]

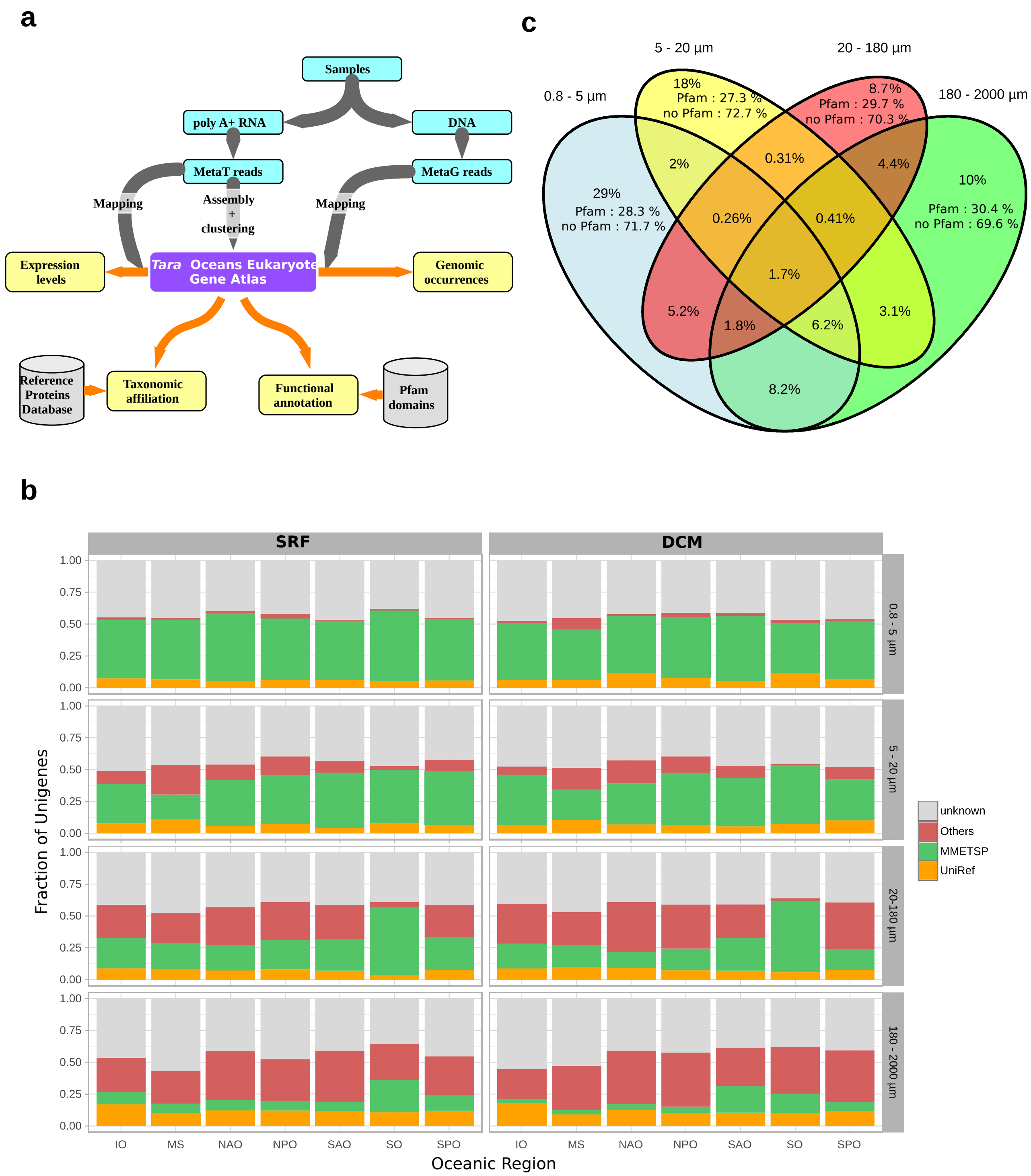

Supplementary Fig. 1: Overview of *Tara* Oceans Eukaryote Gene Catalogue. (a) Summary of methods used to construct and annotate the *Tara* Oceans eukaryote gene catalogue. **(b)** Breakdown of unigenes by oceanic region, depth and size fraction shows widespread gene novelty in the *Tara* Oceans eukaryote gene catalogue. The origin of the best similarity sequence match is indicated as a fraction of the total (MMETSP[1]: release of August 2014, with manual curation; UniRef90[2]: release of September 2014; "Others": are other reference transcriptomes that were generated for alleviating the lack of knowledge in large size fractions, mostly for copepods and rhizaria). Unigenes without significant matches (i.e., those with an e-value > 10^{-5} for their best similarity match) are tagged as "unknown". Note that the MMETSP resource is most useful for identifying genes in the smallest size fractions (likely because of the abundance of picoeukaryote transcriptomes in MMETSP, as well as genes in the 20-180 micron size fraction from the Southern Ocean (likely because of the abundance of diatoms in this oceanic region). **(c)** Distribution of unigenes shows a majority of size fraction-specific sequences. Values indicate the proportion of the catalogue detected by reads originating from one or several different fractions among the 363 samples covering SRF and DCM filters, size fractions 0.8-5, 5-20, 20-180 and 180-2000 µm. For each of the unique classes, the proportion of unigenes bearing a Pfam domain is indicated.

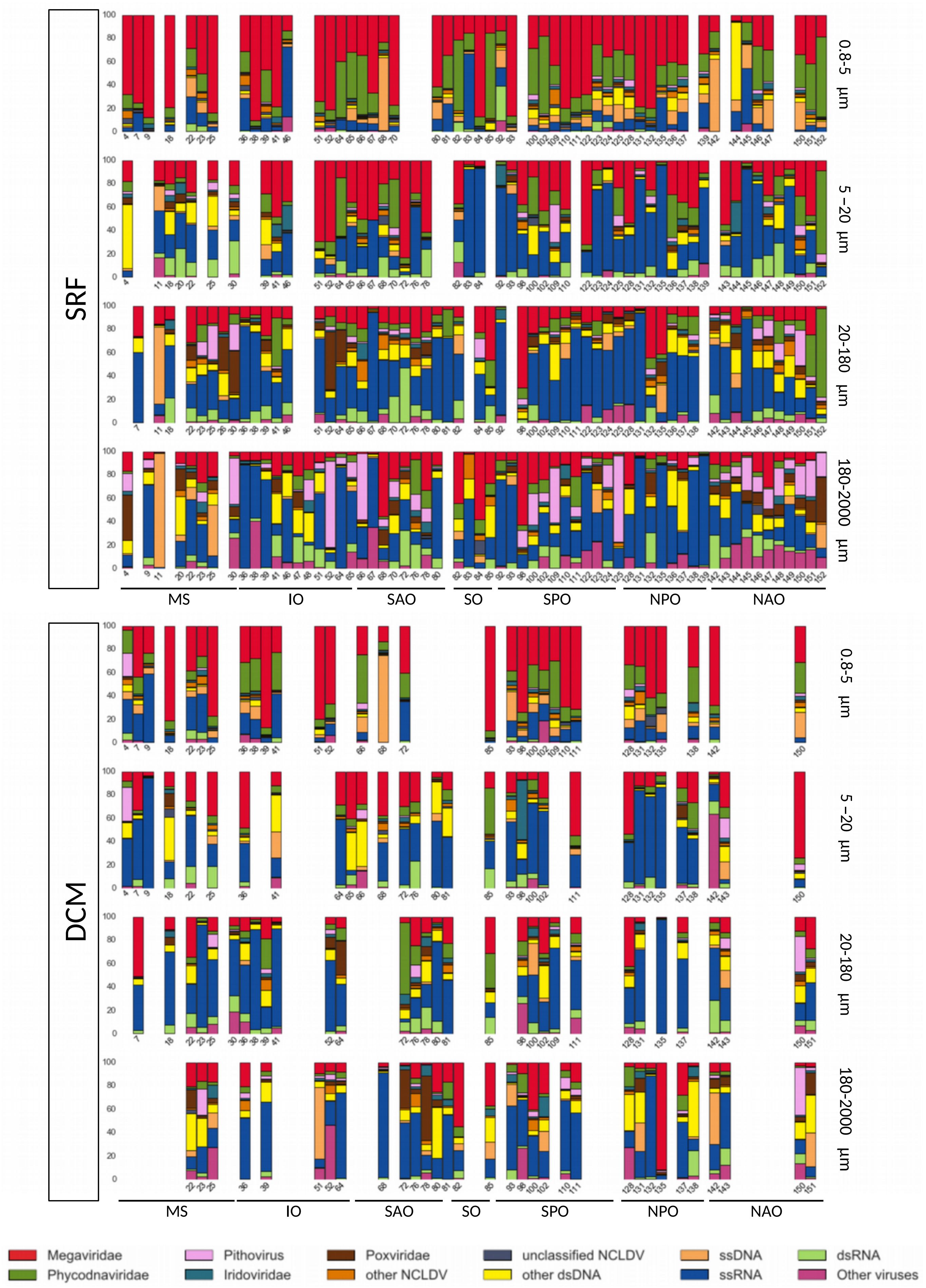

Supplementary Fig. 2: Distribution of viral transcripts and RNA genomes across stations. Relative contributions of different viral groups to the whole eukaryotic viral transcripts (or RNA genomes) are

plotted. Samples are grouped according to sampling depth (SRF or DCM) and size fraction (0.8-5 µm, 5-20 µm, 20-180 µm, 180-2000 µm). Viral compositions largely differ across size fractions, reflecting host-associated detection of their transcripts. For instance, transcripts of *Megaviridae* and *Phycodnaviridae* contribute more than 50% of viral transcripts in 81% (*n*=83) of the samples from the smallest size fractions, whilst RNA virus sequences and to a lesser extend dsDNA other than NCLDV are important in the three largest size fractions. The *Megaviridae* family, which includes amoeba-infecting mimiviruses and other giant viruses infecting heterotrophic flagellates and unicellular algae, represents 70% of the eukaryotic virus unigenes (see Fig. 2c). Notably, out of 23,475 *Megaviridae* unigenes, only 44% (10,216) had more than 50% identity to known *Megaviridae*, suggesting that a significant fraction of the diversity of *Megaviridae* remains to be unravelled. Relative abundance of viral unigenes among all transcripts including those from cellular organisms was one order of magnitude higher in the piconano-planktonic communities (0.04% in average) than in larger size fractions (0.007% in average).

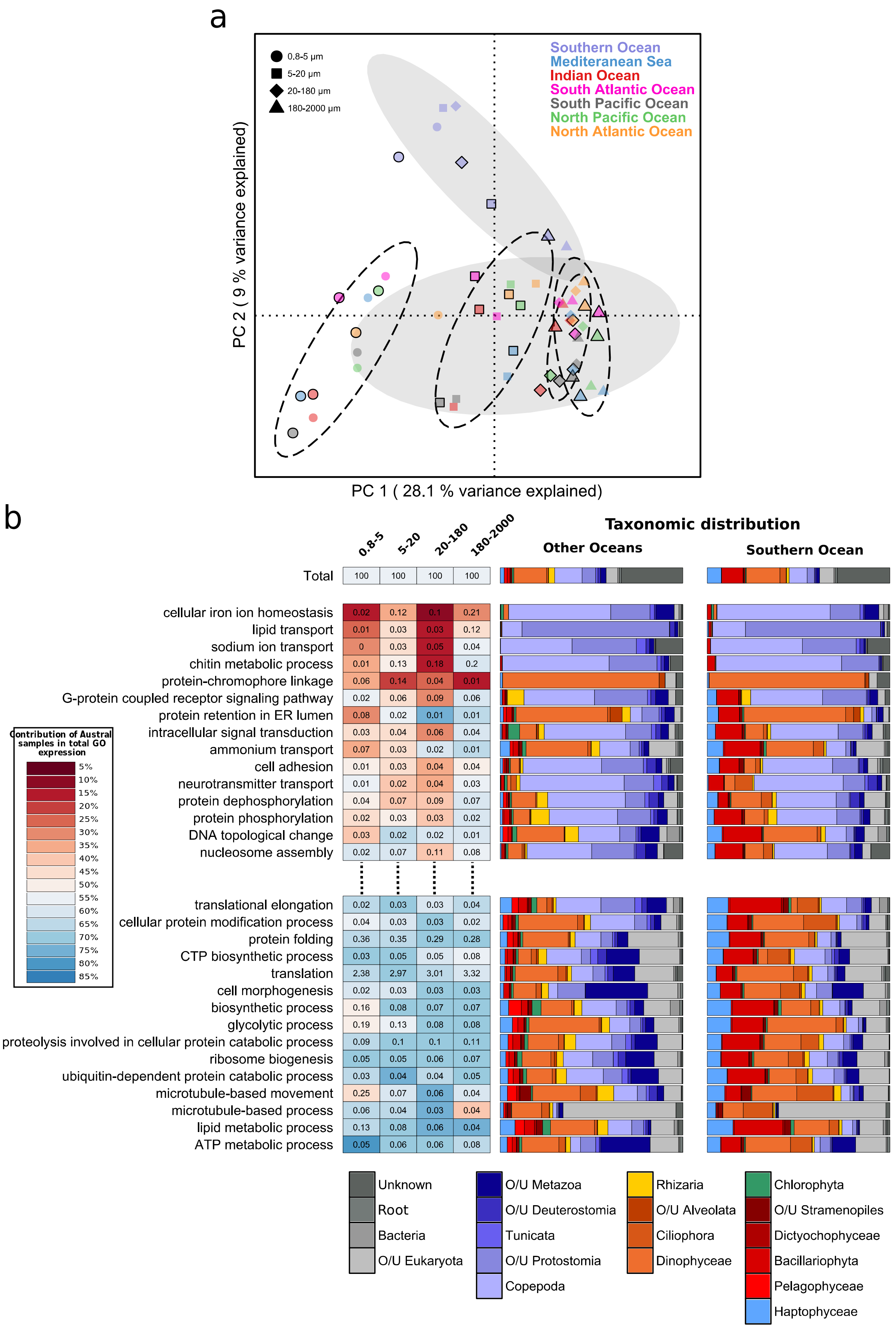

Supplementary Fig. 3: Functional characterization of the gene catalogue. (a) Principal component analysis of the relative expression of all of the unigenes carrying Pfam domains in different oceanic regions. For each size fraction and depth and for each Pfam domain, the relative expression by ocean was obtained by averaging the expression of each sample. The two axes represented explain 37.1% of the variance. *Tara*

Oceans samples are labelled by filter size (symbols), by oceanic region (colour) and by depth (SRF: without contour, DCM: black contour). Confidence ellipses for each size fraction (dashed contour) and Southern Ocean samples (coloured in blue) define regions that contain 85% of samples that can be drawn under a multivariate Student distribution. **(b)** Differentially expressed GO terms and their taxonomic distribution in the Southern Ocean with respect to other ocean provinces. Left panel colour scale (from blue to red) indicates in percentage the average expression of the most expressed GO terms in Southern Ocean (Austral) stations (19 samples) relative to all samples (363 samples). This analysis was performed separately for the main size fractions (4 columns). Relative expression values for Southern Ocean samples are indicated in each rectangle. Right panels: The contribution of each taxonomic group to the total expression of the GO term is shown as an average of all *Tara* Oceans SRF and DCM samples. O/U = Other or Unassigned.

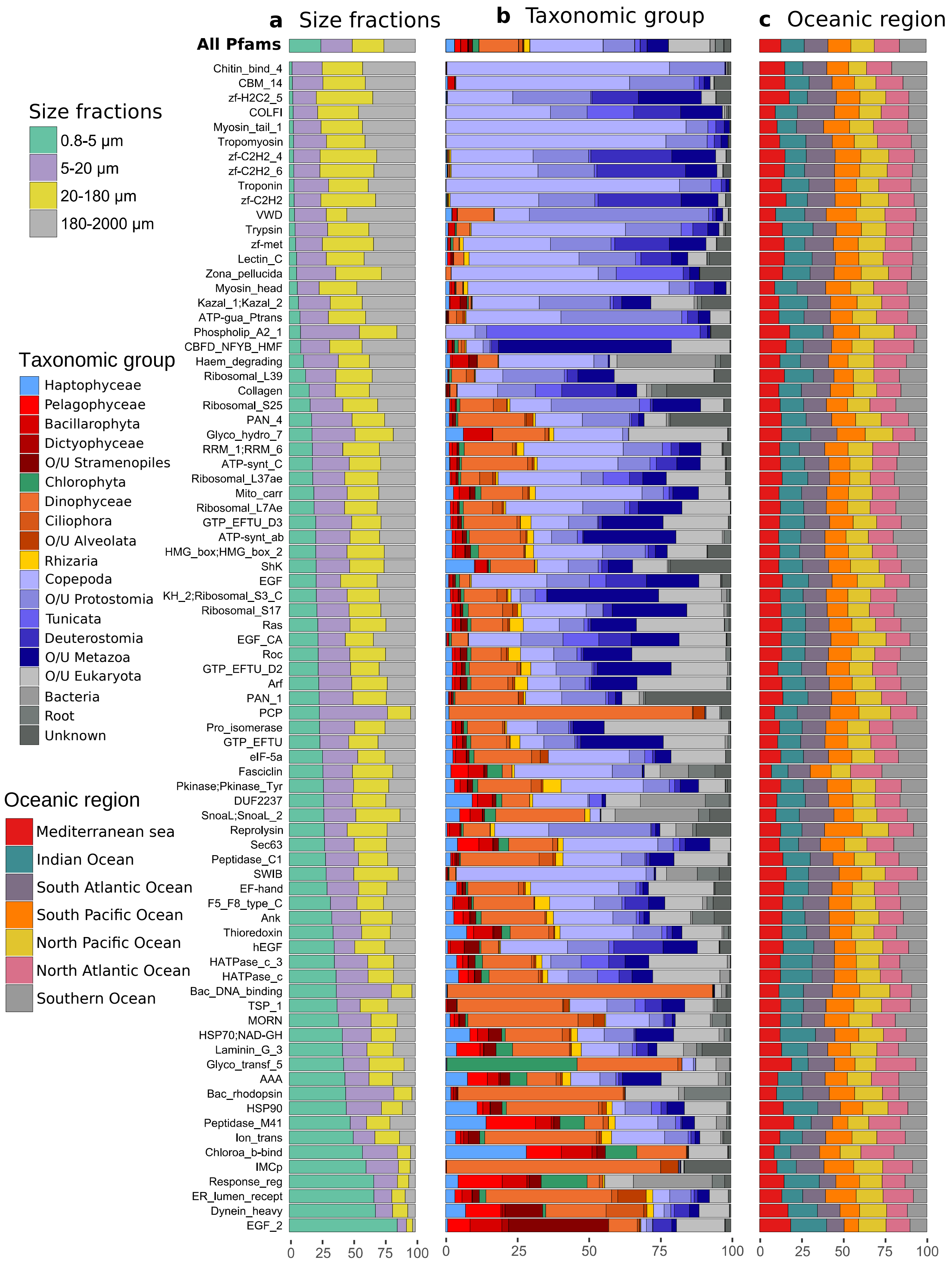

Supplementary Fig. 4: Taxonomic and geographic distributions of most highly expressed Pfam domains. The 50 most expressed Pfam domains from each taxonomic group and each size fraction in all *Tara* Oceans stations (SRF and DCM) have been selected. Among them, 88 are distinct. Highly conserved Pfams with more than 50% of their expression affiliated to "O/U Eukaryota" have been removed (Actin,

CENP-T_C, TAF, Ribosomal_L40e, Rad60-SLD_2, Ubiquitin and Tubulin). (a) Differential relative expression of each Pfam in the four filter sizes. (b) Contribution of each taxonomic group (defined by the taxonomic affiliation of unigenes) to the expression of each Pfam domain. O/U = Other or Unassigned. (c) Differential relative expression of each Pfam in the seven oceanic regions. Detailed scrutiny of the genes containing the EGF-2 domains shows that most of them encode a family of proteins homologous to mastigoneme components SIG/MAS of stramenopiles[3], and are thus also linked to flagellar motility in this group. Bac_DNA binding proteins correspond to the histone-like proteins of dinoflagellates known to have a significant similarity with bacterial DNA binding proteins, suggesting that this highly specific mode of organization of chromatin is ubiquitous among many dinoflagellates sampled in the study. Zinc-finger proteins are highly expressed in animal groups, whilst some protists have a diverse repertoire of genes related to the response regulators of prokaryotes (Response_reg), a family of proteins acting in the response to environmental signals. This family appears to be particularly expressed in pelagophytes, diatoms and chlorophytes. Interestingly, the Pfam domain Von Willebrand factor type D (VWD), typical of multicellular species, seems to be widespread among dinoflagellates as well as haptophytes, suggesting the importance of diverse functions such as cell adhesion, patterning, migration and signal transduction.

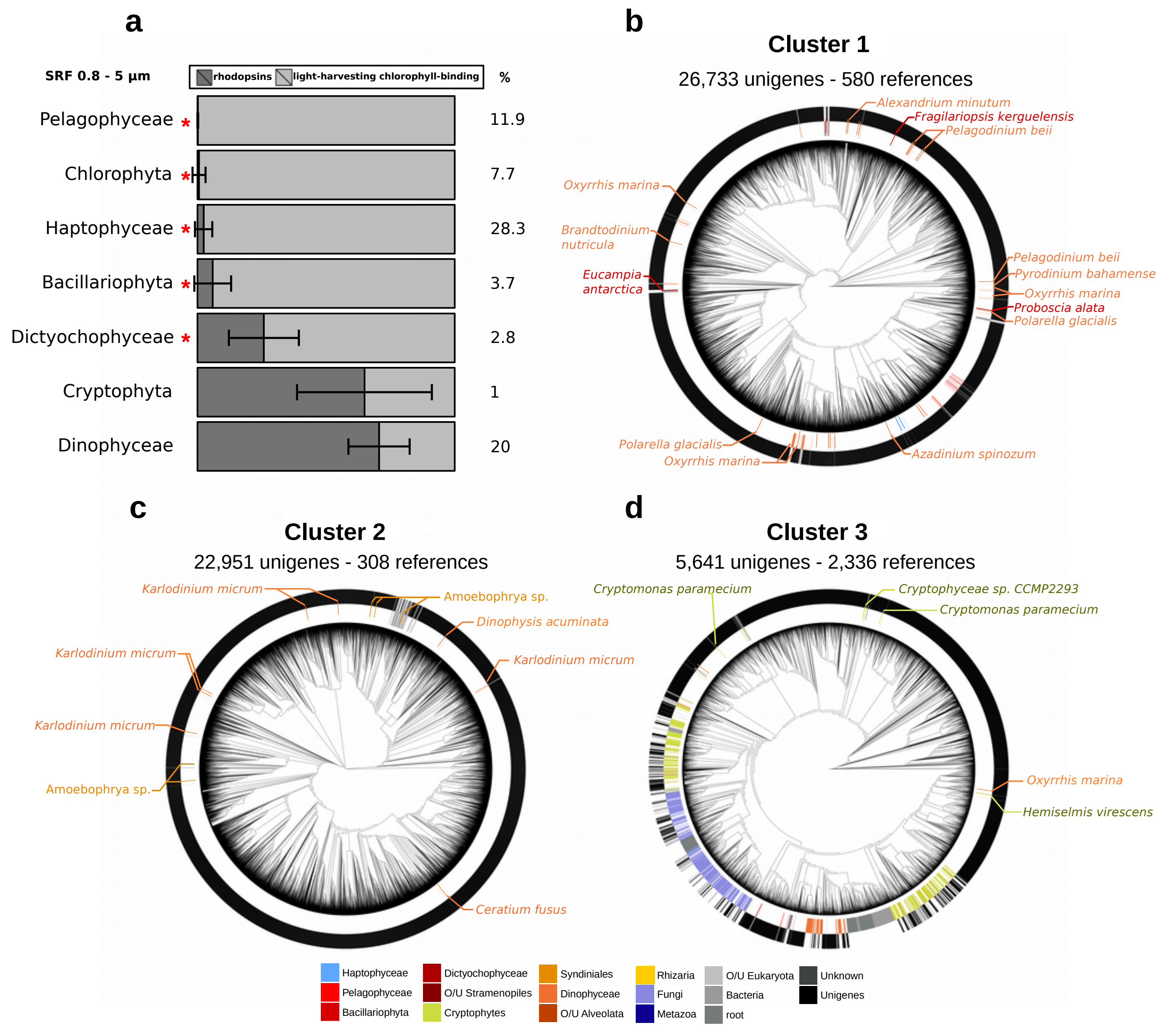

Supplementary Fig. 5: (a) Relative mRNA levels of genes encoding proteorhodopsins and light-harvesting chlorophyll-binding proteins. The relative expression value for each protist taxonomic group in 0.8 – 5 µm filters from surface samples is compared to that of rhodopsin proteins (PF01036, dark grey), which are proposed to use light energy for direct proton pumping. Error bar indicates the standard deviation between samples and red stars indicate that light-harvesting chlorophyll-binding proteins are significantly less expressed than rhodopsin proteins (wilcoxon rank tests with $p<10^{-5}$). The percentage of expression for each taxonomic group is indicated on the right. **(b,c,d) Cladograms from the multiple protein sequence alignments of the 3 largest clusters of type-I rhodopsins (bearing PF01036 motif).** (a) Cluster 1 (b) Cluster 3, (c) Cluster 2. Reference sequences are represented with lines colored according to their taxa (inner ring) whereas unigenes are represented by black lines (outer ring). Names of selected reference organisms are indicated. Translated unigenes (in the Pfam motif frame) and reference sequences were aligned using MAFFT 7.310 and phylogeny was inferred using FastTree 2.1.9 (approximate maximum likelihood).

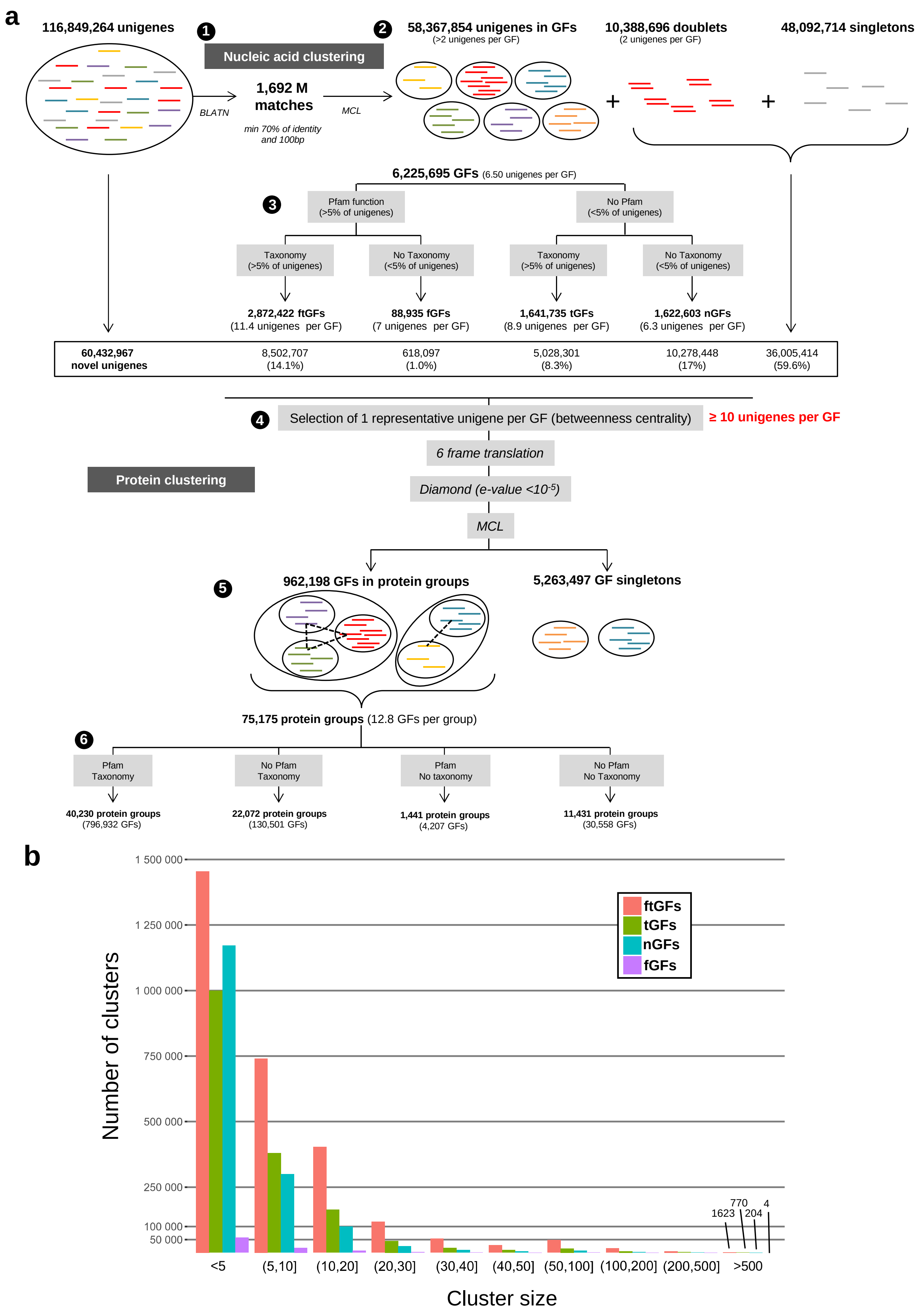

Supplementary Fig. 6: Pipeline and main results from clustering of the eukaryote gene catalogue. (a) Schematic representation of the clustering pipeline (see Methods). **(b)** Size distribution of clusters for each GF category. The y-axis indicates the number of clusters for each cluster size (number of unigenes in the cluster). The number of clusters of more than 500 unigenes is indicated on the top of the bar.

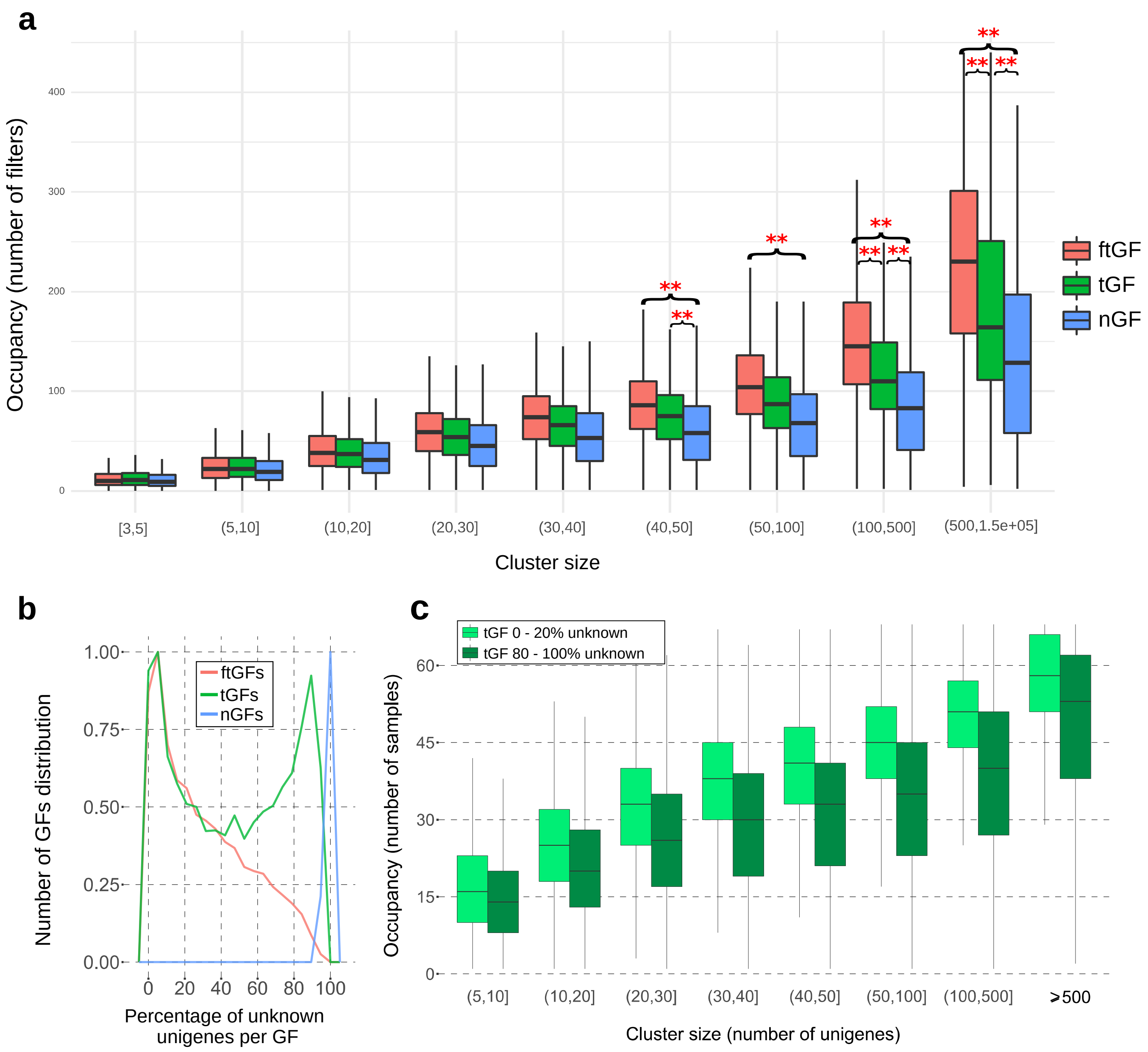

Supplementary Fig. 7: Characterization of gene families encoding proteins with unknown functions. (a) Distribution of GF occupancy for the three main GF categories. GFs are classified according to their size (x-axis). The y-axis indicates the number of samples in which the GF family is expressed (at least one unigene detected with a coverage > 80% of the unigene length). Kolmogorov-Smirnov tests with $p<10^{-5}$ between occupancy distributions are indicated with red stars. **(b)** Density curve of the number of GFs according to their proportion of unknown unigenes for each GF category. **(c)** Distribution of tGF occupancy for tGFs with less than 20% of unknown unigenes (light green) and for tGFs (dark green) with more than 80% of unknown unigenes.

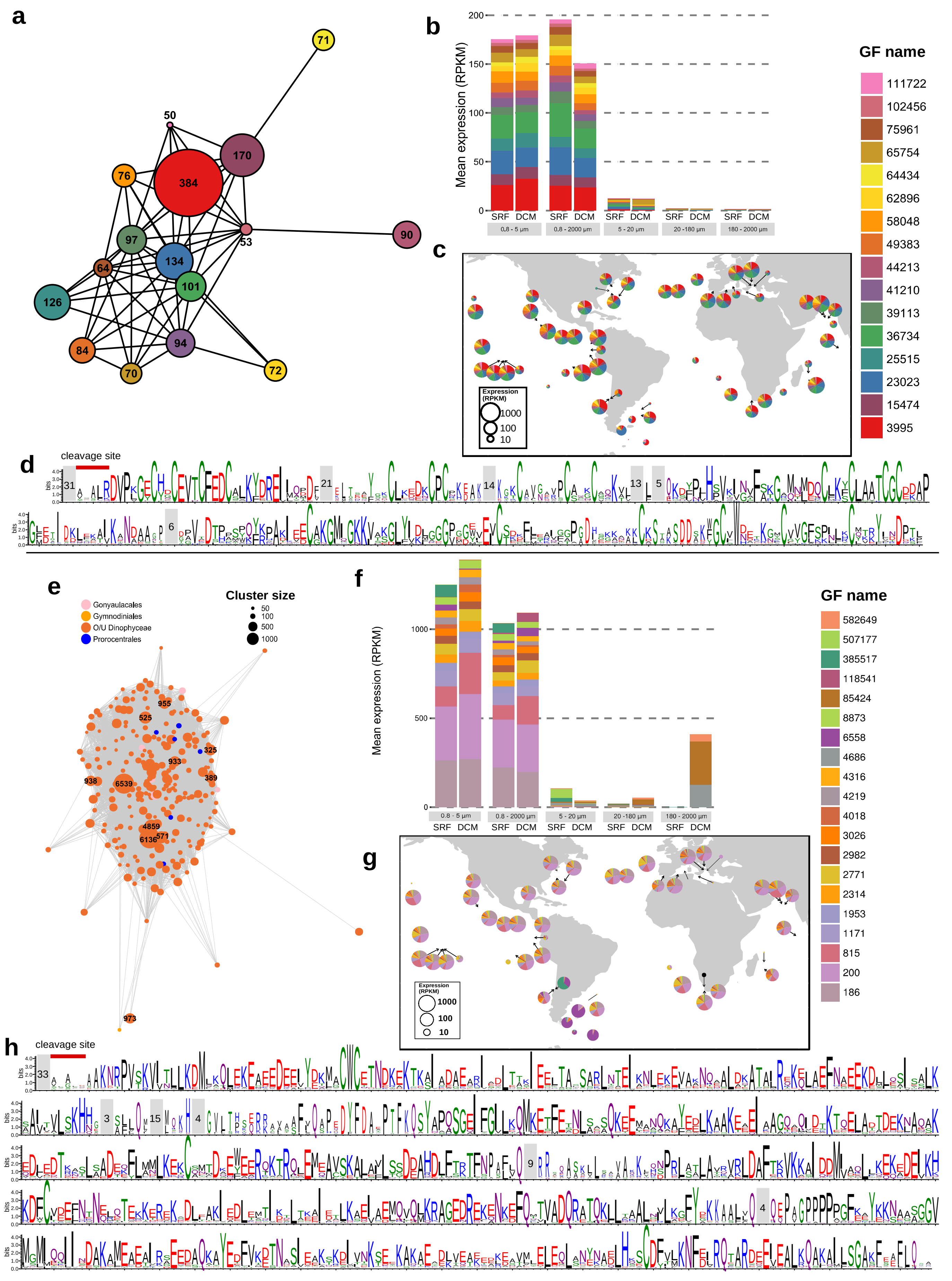

Supplementary Fig. 8: Examples of protein groups expressed in small size fractions. (a-d) New Gene Families expressed in pico-nano size fractions. **(a)** Graph representation of the protein group number *1540*. Each GF of the protein group of more than 50 unigenes is represented by a node with a diameter

proportional to the number of unigenes in the GF. Proteic matches between GFs are represented by an edge. **(b)** Mean expression of GFs in different size-fractions and depths. Each colour corresponds to a GF of protein group *1540*. **(c)** World map representation of protein group *1540* expression in the 0.8 – 5 µm size fraction. SRF and DCM samples have been pooled. Circle diameters represent the relative expression of the protein group in RPKM. The contribution to expression of each GF is represented by the different colours. **(d)** Sequence logo of the multiple alignment of the protein group *1540*. 158 ORFs (343 amino acids in average) of protein group *1540* were aligned and positions with more than 50% of gaps were removed. Mean numbers of amino acids on unaligned regions of the protein are indicated in grey boxes. A cleavage site, indicated on the left part of the sequence logo was predicted on 129 sequences. **(e)** Graph representation of the protein group number 26. Each GF of the protein group of more than 50 unigenes is represented by a node with a diameter proportional to the number of unigenes in the GF. Proteic matches between GFs are represented by an edge. The node colour indicates the taxonomic affiliation of the GF. **(f)** Mean expression of GFs in different size-fractions and depths. Each colour corresponds to a GF of protein group *26*. **(g)** World map representation of GF expression. The circle diameters represent the relative expression of the protein group in RPKM. The contribution to expression of each GF is represented by the different colours. **(h)** Sequence logo of the multiple alignment of the protein group *26*. 376 ORFs (690 amino acids in average) of protein group *26* were aligned and positions with more than 50% of gaps were removed. Mean numbers of amino acids on unaligned regions of the protein are indicated in grey boxes. A cleavage site, indicated on the left part of the sequence logo was predicted on 291 sequences.

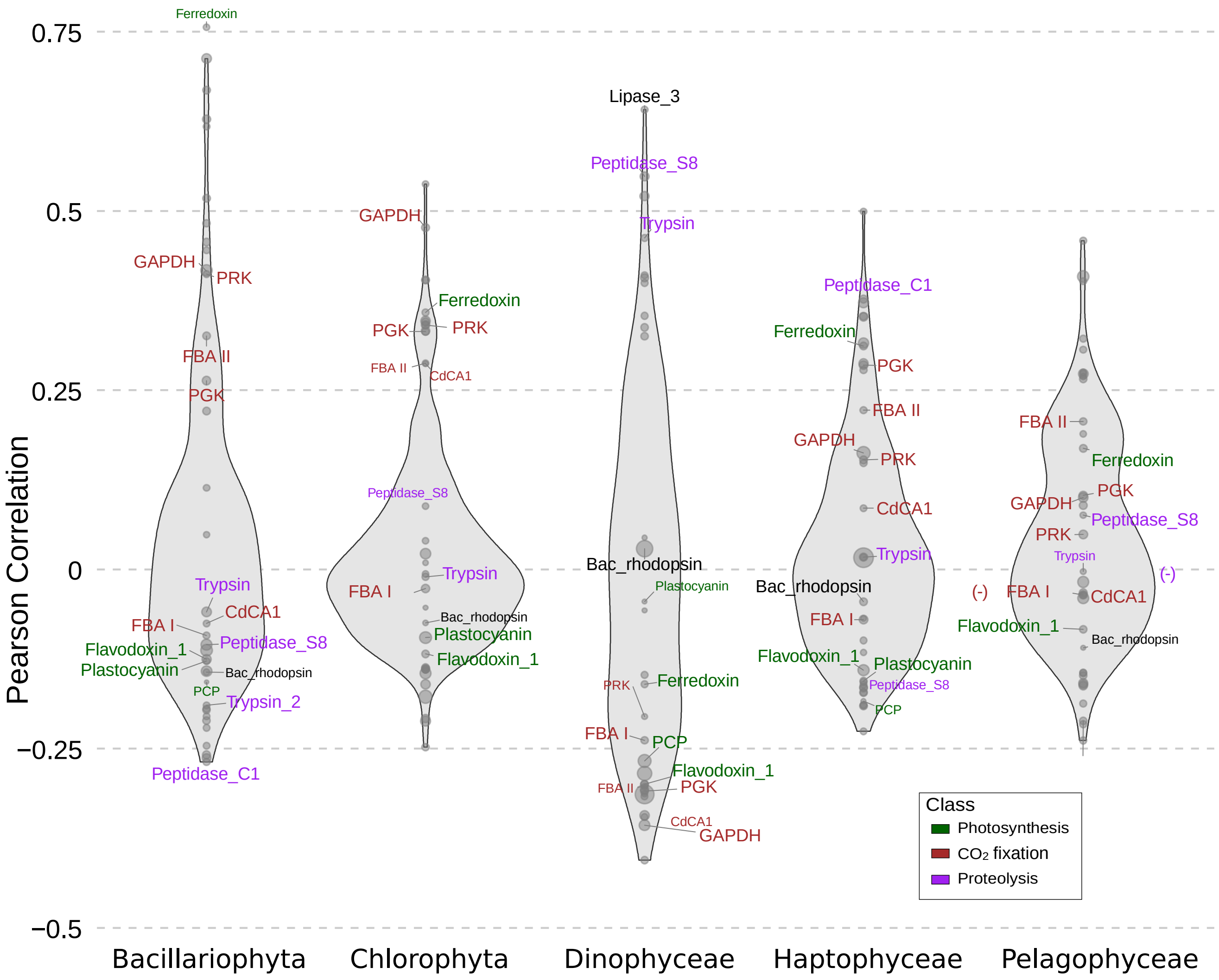

Supplementary Fig. 9: Distribution of Pearson correlation values of Pfam domains with measurements of net primary productivity in the five major photosynthetic planktonic groups. Vertical axis represents the distribution of Pearson correlation parameters of the relative expression of each Pfam domain in each of the five major photosynthetic planktonic groups observed in the size fractions in which they were principally localized (Bacillariophyta [5-20 µm], Chlorophyta [0.8-5 µm], Dinophyceae [5-20 µm], Haptophyceae [0.8-5 µm] and Pelagophyceae [0.8-5 µm]). Pfam expression levels were determined as percentage of the total Pfam expression for that given organismal group in that given size fraction. The profiles illustrate the density distribution of all the correlation values of the Pfams having a relative expression value at least equal to 0.05% (relative expression value is indicated by the size of the grey dots). Pfam representatives of three main functional classes (CO_2 fixation, Photosynthesis and Proteolysis) are coloured. Pfams labelled with a small fontface have a relative expression value smaller than 0.05%. Pfams are shown according to their name codes in the Pfam database, except for: FBA I (Glycolytic) FBA II (F_bP_aldolase), Ferredoxin (Fer2), Plastocyanin (Copper-bind;Cupredoxin_1), and GAPDH (Gp_dh_C;Gp_dh_N). All indicated Pfams have a significant Pearson correlation with net primary productivity (NPP) estimates at each sampling station (two-tailed t-test at 1% error probability), except the five that have the “(-)” symbol.

Supplementary references

1 Keeling, P. J. *et al.* The Marine Microbial Eukaryote Transcriptome Sequencing Project (MMETSP): illuminating the functional diversity of eukaryotic life in the oceans through transcriptome sequencing. *PLoS Biol* **12**, e1001889, doi:10.1371/journal.pbio.1001889 (2014).

2 Suzek, B. E., Wang, Y., Huang, H., McGarvey, P. B. & Wu, C. H. UniRef clusters: a comprehensive and scalable alternative for improving sequence similarity searches. *Bioinformatics* **31**, 926-932, doi:10.1093/bioinformatics/btu739 (2015).

3 Armbrust, E. V. Identification of a new gene family expressed during the onset of sexual reproduction in the centric diatom Thalassiosira weissflogii. *Appl Environ Microbiol* **65**, 3121-3128 (1999).
