# Supplementary material for: A global ocean atlas of eukaryotic genes
Source: Nat Commun. 2018 Jan 25;9:373. doi: 10.1038/s41467-017-02342-1 (PMC5785536; doi:10.1038/s41467-017-02342-1)
Supplement: Supplementary file 2 — Description of Additional Supplementary Files [file 41467_2017_2342_MOESM2_ESM.pdf]

**Description of Additional Supplementary Files**

File Name: Supplementary Data 1
Description: Estimation of the total number of unigenes in samples used to construct the Tara Oceans eukaryote gene catalogue.

File Name: Supplementary Data 2
Description: Evaluation of the redundancy of the Tara Oceans eukaryote gene catalogue.

File Name: Supplementary Data 3
Description: Estimated number of different transcriptomes covered by the catalogue.

File Name: Supplementary Data 4
Description: Estimated number of genes per transcriptome.

File Name: Supplementary Data 5
Description: Sampling and Parameters

File Name: Supplementary Data 6
Description: Tara Oceans metatranscriptomic reads assembly

File Name: Supplementary Data 7
Description: Associated Pfams. List of Pfam groups (1st column) that have been detected on the same unignes in more than 30% of cases (last column).

File Name: Supplementary Data 8
Description: BWA mapping resulsts of Metagenomics and Metatranscriptomics reads on the gene catalog.
